# Molecular structures of cdc2-like kinases in complex with a new inhibitor chemotype

**DOI:** 10.1371/journal.pone.0196761

**Published:** 2018-05-03

**Authors:** Anne Walter, Apirat Chaikuad, Renate Helmer, Nadège Loaëc, Lutz Preu, Ingo Ott, Stefan Knapp, Laurent Meijer, Conrad Kunick

**Affiliations:** 1 Institut für Medizinische und Pharmazeutische Chemie, Technische Universität Braunschweig, Braunschweig, Germany; 2 Center of Pharmaceutical Engeneering (PVZ), Technische Universität Braunschweig, Braunschweig, Germany; 3 Structural Genomics Consortium, Nuffield Department of Medicine, Oxford University, Oxford, United Kingdom; 4 Institute for Pharmaceutical Chemistry, Structural Genomics Consortium and Buchmann Institute for Molecular Life Sciences, Johann Wolfgang Goethe-University, Frankfurt am Main, Germany; 5 ManRos Therapeutics, Hôtel de Recherche, Centre de Perharidy, Roscoff, France; University of Parma, ITALY

## Abstract

Cdc2-like kinases (CLKs) represent a family of serine-threonine kinases involved in the regulation of splicing by phosphorylation of SR-proteins and other splicing factors. Although compounds acting against CLKs have been described, only a few show selectivity against dual-specificity tyrosine phosphorylation regulated-kinases (DYRKs). We here report a novel CLK inhibitor family based on a 6,7-dihydropyrrolo[3,4-*g*]indol-8(1*H*)-one core scaffold. Within the series, 3-(3-chlorophenyl)-6,7-dihydropyrrolo[3,4-*g*]indol-8(1*H*)-one (KuWal151) was identified as inhibitor of CLK1, CLK2 and CLK4 with a high selectivity margin towards DYRK kinases. The compound displayed a potent antiproliferative activity in an array of cultured cancer cell lines. The X-ray structure analyses of three members of the new compound class co-crystallized with CLK proteins corroborated a molecular binding mode predicted by docking studies.

## Introduction

Splicing is a major regulator in the process of protein biosynthesis during which pre-mRNA is modified at the spliceosome. Introns are removed and exons reconnected within the nucleus to form the final mRNA which is then translocated to the cytoplasm and translated into the respective amino acid sequence at the ribosomes. By reconnecting different exons, multiple mRNA sequences are generated originating from the same gene leading to different isoforms of a specific protein, with different and sometimes opposing functions. This alternative splicing process is tightly regulated by various proteins, such as protein kinases, and is observed for approximately 95% of genes consisting of multiple exons [1,2]. Dysregulation in splicing has been identified as a relevant contributor to the development of diseases [3], e.g. neurodegenerative disorders such as Alzheimer’s disease [4,5]. Alternative splicing is often a regulatory mechanism which may result in translation of proteins with different function. For instance, splicing of vascular epithelial factor is regulated by kinases of the SRPK family. The inhibition of SRPK1 results in predominant expression of antiangiogenic VEGF isoforms, a phenotypic response that has been exploited for the development of SRPK inhibitors for treatment of macular degeneration [6,7].

In various cancers, alternative splicing has been shown to contribute to survival of tumor cells by suppression of apoptosis [8], to cancer cell migration and adhesion [9], and to resistance against antitumor drugs [10,11]. In this context, the use of small molecules modulating the splicing machinery has been suggested as therapeutic option in cancer therapy [12].

The human splicing factor SPF45 is overexpressed in various cancer tissues and is involved in the formation of drug-resistant tumor cells [13]. The protein kinase CLK1 (cdc2-like kinase1) modulates activity of SPF45 by regulating its expression and by phosphorylating the protein at distinct serine residues [14]. The observation that siRNA-induced knockdown of CLK1 leads to SPF45 degradation suggests the use of small molecular CLK1 inhibitors as anticancer drugs [14].

The CLK protein kinase family is closely related both in structure and function to the family of dual-specificity tyrosine phosphorylation-regulated kinases (DYRKs). Sequence homology between CLK1 (PDB-ID: 1Z57) and DYRK2 (PDB-ID: 4AZF) was determined to be 55% (36% identity) according to the Blast algorithm applied through the protein data bank interface [15].

**Fig 1:**
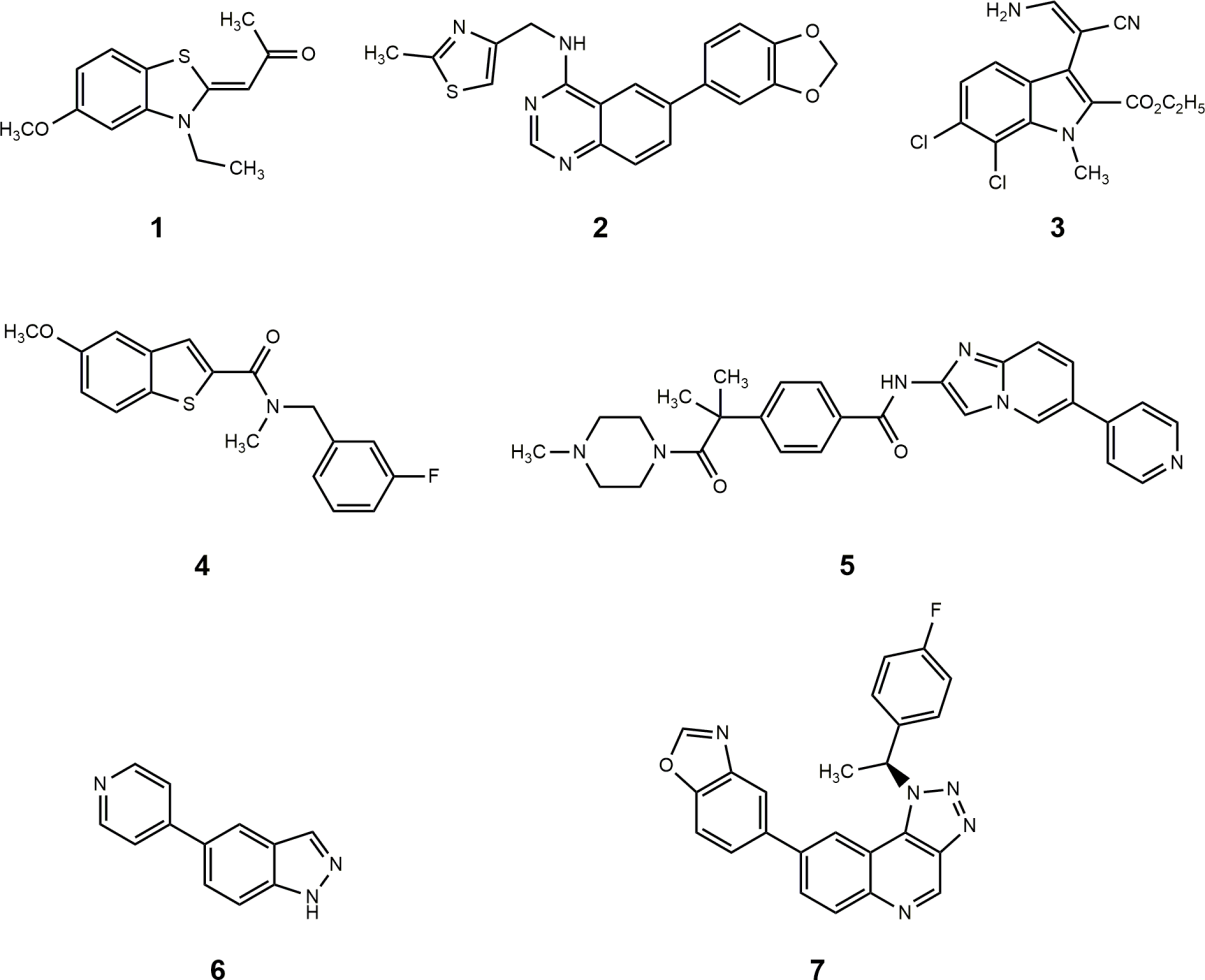
CLK1 inhibitors described in the literature. TG003 (**1**); NCGC00185963 (**2**), KH-CB19 (**3**); benzo[*b*]thiophen-2-carboxamide **4**; T3 (**5**); TG693 (**6**); [1,2,3]triazolo[4,5-*c*]quinoline **7**.

The DYRK kinases, especially DYRK1A, have been associated with mental retardation in Down syndrome as well as Alzheimer’s disease and are also involved in the regulation of splicing [16–19]. If inhibitors are used in biochemical assays to study the role of either CLKs or DYRKs, selective reagents are needed for an accurate differentiation between the specific kinase effects. The ATP binding pockets of CLK1 and DYRK1A are rather similar both in terms of sequence and surface shape. In both kinases the gatekeeper (gk) residue is a phenylalanine, and also the three following amino acids are identical (gk+1; gk+3) or similar (gk+2: leucine in CLK1 and methionine in DYRK1A). Given the structural similarity of the two kinase families, the design of specific and potent ATP-competitive inhibitors is a challenging task. Several small molecules (Fig 1) have been reported as concomitant inhibitors of both the CLK and the DYRK kinase families [20,21]. The CLK inhibitor TG003 (**1**) [22] was frequently used in biological studies. In a kinase binding assay reported by Mott et al., TG003 showed comparable K_d_ values against CLK1 (19 nM) and against DYRK1A (12 nM) [23]. Within a series of quinazoline derivatives, the compound NCGC00185963 (**2**) was reported to exhibit moderate selectivity for CLK1 versus DYRK1A (IC_50_ CLK1: 96 nM; DYRK1A 206 nM). A kinome wide binding assay showed that **2** interacted only with protein kinases of the CMGC family [24]. The potent CLK1 inhibitor KH-CB19 (**3**) displayed also activity against DYRK1A (IC_50_ CLK1: 20 nM; DYRK1A 55 nM) [25]. Until recently it was a matter of debate whether the design of truly selective inhibitors for either kinase would be possible. In 2015 the first potent DYRK1A inhibitor with substantial selectivity versus CLK1 was published [26]. The benzo[*b*]thiophen-2-carboxamide **4** was reported as potent CLK1 inhibitor with considerable selectivity versus DYRK1A (IC_50_ CLK1: 7 nM; DYRK1A 340 nM) [27]. Consistent with the modulation of SP45 activity by CLK1 and earlier findings that CLK inhibitors induce apoptosis and show antitumor activity in vitro [28], **4** inhibited the growth of certain cancer cell lines at submicromolar concentrations [27]. Compound T3 (**5**) was disclosed as a subnanomolar CLK1 inhibitor, displaying selectivity versus DYRK1A of more than three orders of magnitude (IC_50_ CLK1: 0.67 nM; DYRK1A 260 nM). In line with its potent CLK inhibition, **5** inhibited the growth of a colorectal cancer cell line at submicromolar concentrations (GI_50_ HCT116: 130 nM) [29]. The CLK inhibitor TG693 (**6**) modulates the mutated exon 31 of the dystrophin gene in mice, and might be a suitable starting point for the development of therapeutics against the Duchenne muscular dystrophy (DMD). 1 μM TG693 inhibited the activities of DYRK1A by 84% and of CLK1 by 93%, exhibiting some selectivity for the latter enzyme [30]. Recently, the [1,2,3]triazolo[4,5-*c*]quinoline **7** was reported as potent and selective CLK1 inhibitor, inducing autophagy *in vitro* and showing hepatoprotective effects *in vivo* in an acetaminophen-induced liver injury mouse model [31]. We here report 6,7-dihydropyrrolo[3,4-*g*]indol-8(1*H*)-ones as another class of CLK inhibitors. A distinct representative of this compound family, 3-(3-chlorophenyl)-6,7-dihydropyrrolo[3,4-*g*]indol-8(1*H*)-one (KuWal151, **8c**), exhibited selectivity versus DYRK1A and other DYRK kinases and showed potent antiproliferative activity against a broad panel of in vitro cultured cancer cell lines.

## Screening campaign for CLK inhibitors

**Fig 2:**
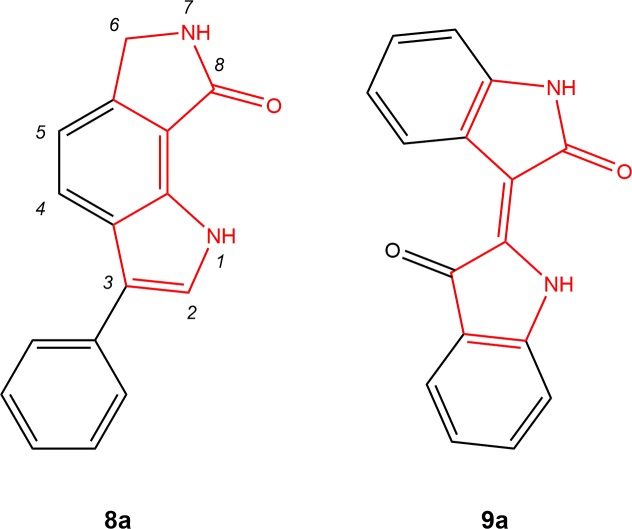
Hit compound 8a identified as CLK1 inhibitor in a library screening and the structurally related dye indirubin (9a). Both compounds comprise a pyrrole unit connected to a γ-butyrolactam ring via a double bond.

The novel protein kinase inhibitor family of 6,7-dihydropyrrolo[3,4-*g*]indol-8(1*H*)-ones was discovered during a screening campaign of an in house compound library for CLK inhibition by radiometric *in vitro* kinase assays. In order to assess selectivity, activities on other kinases of the CMGC group besides CLKs (CDK1/cyclin B, CDK2/cyclin A, CDK5/p25, CDK9/cyclin T, CK1, DYRK1A, DYRK1B, DYRK2, DYRK3, GSK-3β) were tested as well. While the 3-substituted derivative **8a** was identified as a slightly selective inhibitor of CLK1, some 2,3-disubstituted congeners and a 6-oxo derivative were less active or not as selective versus casein kinase 1 (CK1) and against DYRKs (S1 Table). To our best knowledge, 6,7-dihydropyrrolo[3,4-*g*]indol-8(1*H*)-ones like **8a** unsubstituted at the 6- or 7-position have not been published before. Comprising a pyrrole unit connected to a γ-butyrolactam ring via a double bond, **8a** is structurally related to the GSK-3/CDK inhibitor indirubin (**9a**) [32–34](Fig 2).

## Molecular docking

**Fig 3:**
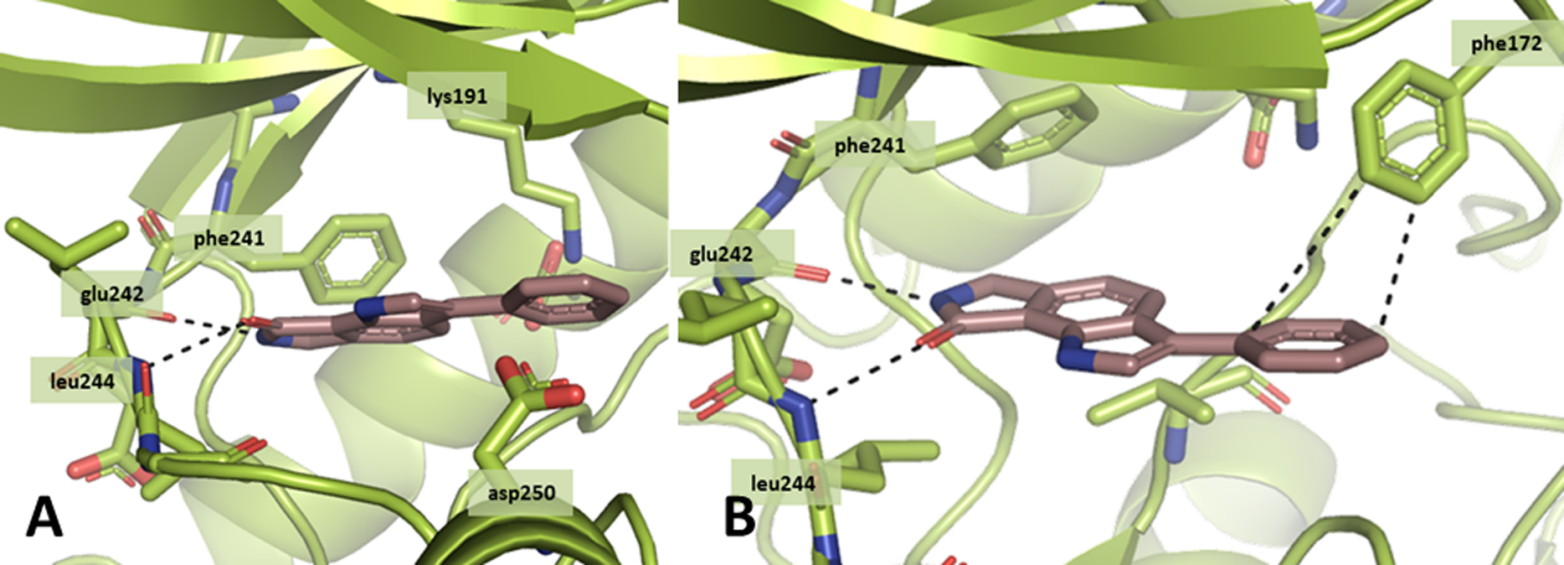
Results of a docking experiment with 8a in CLK1 (PDB-ID: 1Z57). A: front view; B: top view; dashed lines: H-bonds and edge to face interaction.

Via *in silico* molecular docking, candidates for the synthesis of **8a**-related derivatives were designed based on the predicted binding mode of the basic heterocyclic scaffold in the ATP-binding pocket of CLK1 (PDB-ID: 1Z57). The docking tool GOLD [35] was used to fit the inhibitor **8a** into the ATP binding pocket of a published CLK1 crystal structure (PDB-ID: 1Z57 [36]) (Fig 3). Based on this prediction, the pyrrolinone moiety of **8a** is oriented towards the hinge region forming two hydrogen bonds, one being established between gk+1 (Glu242) and the NH of the ligand and a second via the carbonyl oxygen to Leu244 (gk+3). The indole nitrogen is not involved in direct hydrogen bonding to the hinge region. The planar heterocyclic core scaffold is positioned in the adenine pocket of the binding site. At the entrance of the ATP pocket, the 3-phenyl substituent is situated establishing an edge to face interaction [37] with Phe172 of the p-loop. From the top view it becomes visible that the binding site is not filled completely by **8a,** offering further possibilities for additional hydrogen bonding, for example to Asp250 which could be addressed by polar substituents at the phenyl ring. Moreover, there is some unoccupied space in the back of the binding site towards the gatekeeper Phe241 which could be filled by substituents of moderate size at position 5.

Based on the outcome of the docking studies with **8a**, analogues were designed with the intention of creating ligands with improved CLK inhibitory potency and selectivity versus other kinases. For example, docking of the 3-hydroxyphenyl derivative **8g** predicted the formation of a hydrogen-bond between the hydroxyl group and Asp250 of CLK1, so that an increase in affinity was expected. Introduction of halogens at position 5 of the parent ring system led to analogues **12a-c**, which were predicted to occupy previously unused space in the binding pocket. Larger substituents in the 5-position (analogues **17a-c**) appeared too big for this area, but were also prepared for means of comparison. Alkylation at the indole nitrogen with short chains did not alter the predicted binding mode and were introduced with the aim to enable additional contacts with the protein. On the other hand, a substitution at the nitrogen in position 7 led to derivatives for which the docking studies were unable to reproduce the binding mode suggested for **8a** and which were expected to show reduced kinase inhibitory activity.

## Chemistry

**Fig 4:**
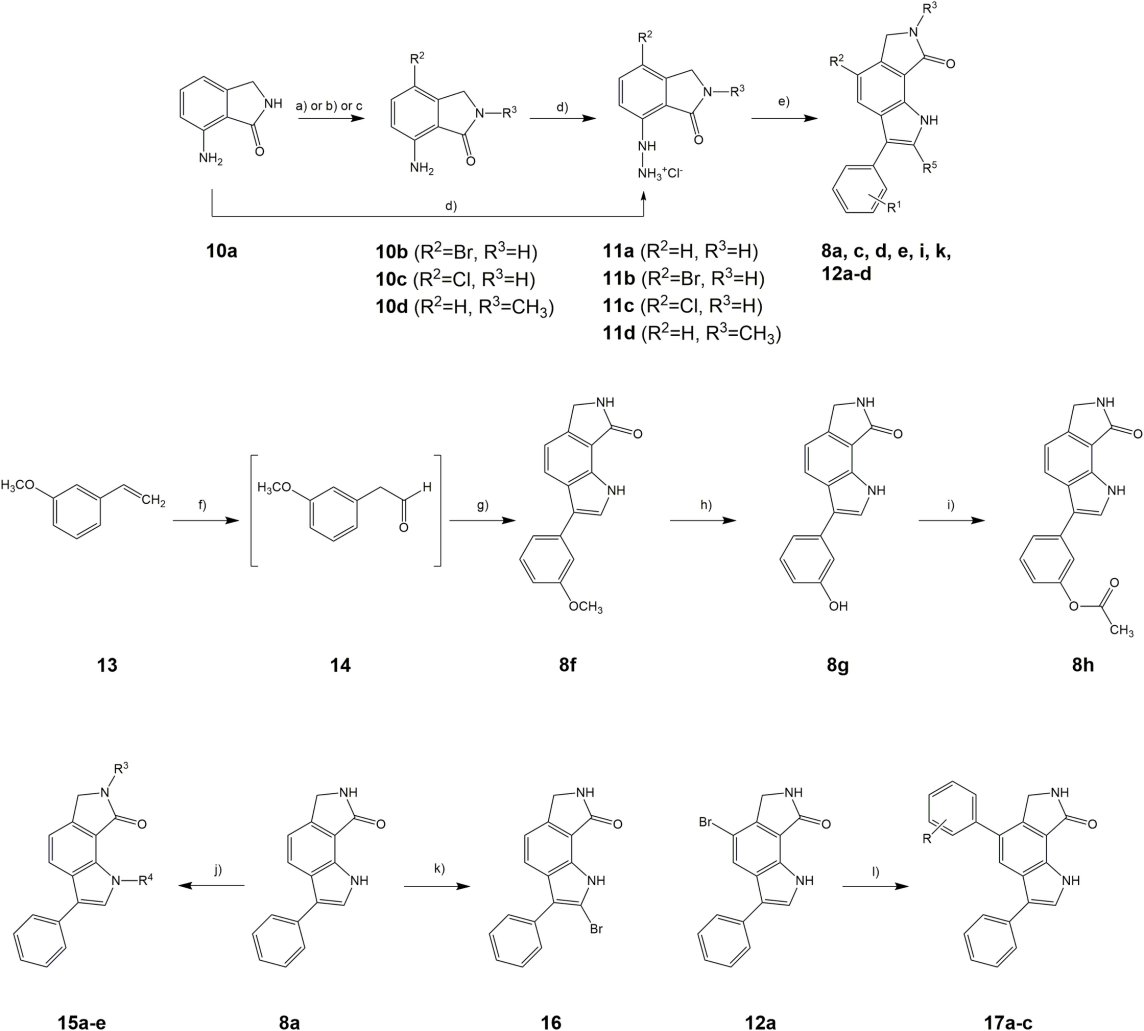
Synthesis procedures for 3-aryl-6,7-dihydropyrrolo[3,4-*g*]indol-8(1*H*)-ones. For residues R^**1**^-R^**5**^ refer to Table 1. Reagents and conditions: (reagents and conditions a, for synthesis of **10b**) NBS, CH_**2**_Cl_**2**_, - 8°C, 1 h, 78%; (reagents and conditions b, for synthesis of **10c**) NCS, acetonitrile, 60°C → reflux, 2 h, 81%; (reagents and conditions c, for synthesis of **10d**) CH_**3**_I, KO*t*Bu, THF, RT, N_**2**_, 24 h, 54%; (d) 1. 37% HCl, NaNO_**2**_, < 0°C; 2. 37% HCl, SnCl_**2**_ x 2 H_**2**_O, 30 min; (e) aldehyde or ketone or acetal, acetic acid, 95°C, 3.5 h, 10%-31%; (f) PdCl_**2**_(MeCN)_**2**_, *p*-benzoquinone, *tert*-butanol, water, 80°C; (g) **11a**, ethanol, H_**2**_SO_**4**_, H_**2**_O, 50°C, 2.5 h, 23%; (h) BBr_**3**_, CH_**2**_Cl_**2**_, RT, N_**2**_, 1 h, 33%; (i) acetic anhydride, pyridine, 4-DMAP, RT, 3 h, 29%; (j) alkyl halide, KO*t*Bu, acetone, RT, N_**2**_, 24 h, 15%-48%; (k) NBS, CH_**2**_Cl_**2**_/acetic acid, < 10°C, N_**2**_, 1.5 h, 29%; (l) appropriate arylboronic acid, Cs_**2**_CO_**3**_, toluene/ethanol, mircowaves, 150°C, 20 min, 5.4%-31%.

**Fig 5:**
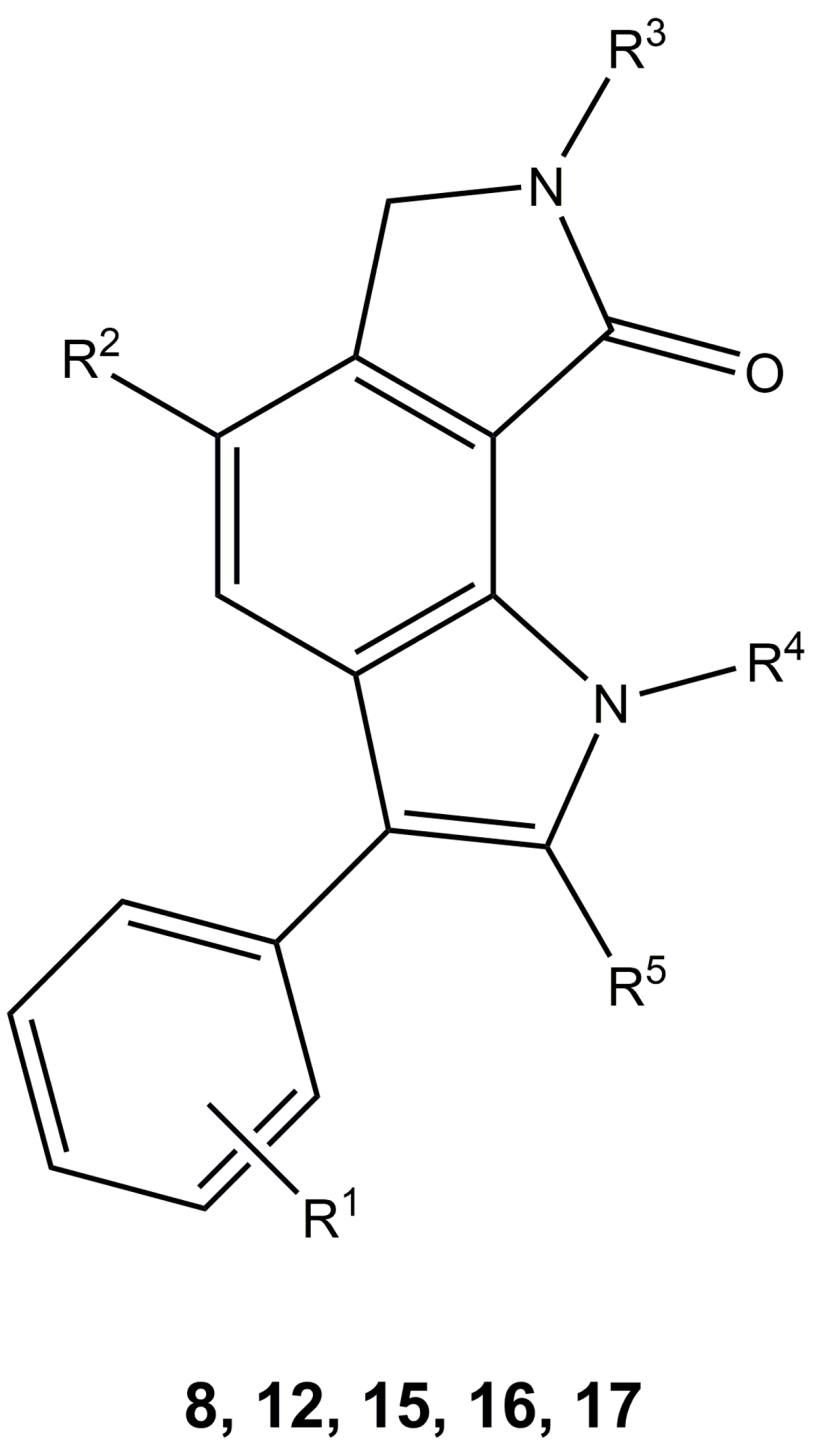
3-Aryl-6,7-dihydropyrrolo[3,4-*g*]indol-8(1*H*)-ones listed in Table 1.

Starting from commercially available 7-aminoisoindolin-1-one **10a**, the arylhydrazines **11a-d** were prepared as central building blocks for the construction of the 6,7-dihydropyrrolo[3,4-*g*]indol-8(1*H*)-one core scaffold. Introduction of halogen substituents at position 4 of the 7-aminoisoindolin-1-one was achieved by reaction with *N*-bromosuccinimide (NBS) or *N*-chlorosuccinimide (NCS), respectively. A selective methylation of **10a** at the pyrrolidinone nitrogen was accomplished with iodomethane and potassium *tert*-butylate in THF furnishing **10d** [38]. Reaction of **10a-d** with sodium nitrite and hydrochloric acid yielded the corresponding diazonium chlorides which were directly reduced to the hydrazinium chlorides **11a-d**. Heating in acetic acid with suitable aldehydes or ketones yielded the 3-phenyl-6,7-dihydropyrrolo[3,4-*g*]indol-8(1*H*)-one derivatives **8** as products of a Fischer indole reaction. Due to the formation of numerous side products, extensive purification processes were necessary to obtain clean products which in turn led to low yields. Especially the 2-phenylacetaldehyde derivatives proved to be very unstable during the reaction as well as under storage conditions. Aliphatic aldehydes in general are known to form adducts or polymers by self-condensation. It has been previously described that 2-phenylacetaldehydes are particularly prone to degradation because of the activated methylene group between carbonyl group and aromatic ring [39]. Therefore, the commercially available dimethyl acetal of 2-phenylacetaldehyde was used as a more stable substitute for the free aldehyde in the synthesis of **8a**. As exemplified for the synthesis of **8f**, the adaption of a method published by Grubbs *et al*. [40] offered the possibility to avoid handling of the aldehydes by oxidation of styrenes, directly reacting the *in situ*-formed aldehydes in the Fischer indole ring closure. The phenol derivative **8g** was prepared by boron tribromide-provoked ether cleavage of the methoxy derivative **8f** [41]. Subsequent treatment with acetic acid anhydride yielded the ester **8h**. Alkyl substituents at the indole nitrogen were introduced after formation of the indole ring system by reaction of alkyl halides with **8a** in the presence of potassium *tert*-butylate using acetone as solvent. The choice of solvent is essential for a selective alkylation at the indole nitrogen in the presence of secondary lactam groups [38]. However, methylation at both nitrogen atoms of **8a** was observed when iodomethane was employed as alkylating reagent, leading to the formation of side product **15e** in significant amounts. The 2-brominated derivative **16** was prepared by reaction of **8a** with *N*-bromosuccinimide. Eventually, the 5-aryl substituted derivatives **17a-c** were synthesized by a microwave-assisted Suzuki-Miyaura coupling of **12a** with arylboronic acids (Fig 4) [42]. The compounds prepared by these methods are represented by the general formula depicted in Fig 5 and are listed in Table 1.

**Table 1:** Structures of and CMGC kinase inhibition by 3-phenyl-6,7-dihydropyrrolo[3,4-*g*]indol-8(1*H*)-ones (IC_50_-values [μM])^a^.

	R^1^	R^2^	R^3^	R^4^	R^5^	CDK1/cyclin B	CDK2/cyclinA	CDK5/p25	CDK9/cyclin T	CK1	CLK 1	CLK 2	CLK 3	CLK 4	DYRK 1A	DYRK 1B	DYRK 2	DYRK 3	GSK3
**8a**	H	H	H	H	H	> 10	> 10	> 10	> 10	> 10	**0.046**	**0.21**	> 10	**0.12**	> 10	4.8	**0.72**	> 10	> 10
**8b**	2-Cl	H	H	H	H	> 10	> 10	> 10	> 10	> 10	**0.14**	nd	nd	nd	1.1	nd	nd	nd	> 10
**8c**	3-Cl	H	H	H	H	> 10	> 10	> 10	> 10	> 10	**0.088**	**0.51**	> 10	**0.028**	> 10	> 10	> 10	> 10	> 10
**8d**	4-Cl	H	H	H	H	> 10	> 10	> 10	> 10	> 10	**0.10**	**0.17**	1.8	**0.13**	> 10	> 10	9.3	> 10	> 10
**8e**	3,4-dichloro	H	H	H	H	> 10	> 10	> 10	> 10	> 10	**0.11**	**0.69**	> 10	**0.13**	> 10	> 10	> 10	> 10	> 10
**8f**	3-OCH_3_	H	H	H	H	> 10	> 10	> 10	> 10	> 10	**0.11**	**0.32**	2.8	**0.11**	3.8	1.9	2.1	5.4	> 10
**8g**	3-OH	H	H	H	H	> 10	> 10	> 10	1.1	> 10	**0.044**	**0.082**	1.7	**0.081**	**0.74**	**0.39**	**0.59**	2.2	> 10
**8h**	3-OCOCH_3_	H	H	H	H	> 10	> 10	> 10	> 10	> 10	**0.17**	**0.32**	3.3	**0.13**	1.4	1.5	2.4	6.1	> 10
**8i**	3-CH_3_	H	H	H	H	> 10	> 10	> 10	> 10	> 10	**0.061**	**0.41**	5.1	**0.14**	2.6	4.1	3.1	> 10	> 10
**8j**	3-NO_2_	H	H	H	H	> 10	> 10	> 10	> 10	> 10	**0.038**	**0.37**	8.5	**0.061**	> 10	1.5	4.9	1.9	> 10
**8k**	H	H	H	H	CH_3_	> 10	> 10	> 10	> 10	> 10	**0.093**	nd	nd	nd	1.5	nd	nd	nd	> 10
**8l**	H	H	H	H	C_2_H_5_	> 10	> 10	> 10	> 10	6.1	**0.12**	nd	nd	nd	**0.81**	nd	nd	nd	> 10
**12a**	H	Br	H	H	H	2.8	> 10	5.3	> 10	1.7	**0.043**	nd	nd	nd	**0.20**	nd	nd	nd	> 10
**12b**	H	Br	H	H	CH_3_	> 10	> 10	> 10	> 10	> 10	**0.023**	**0.025**	7.1	**< 0.03**	**0.11**	**0.11**	**0.11**	**0.12**	> 10
**12c**	H	Cl	H	H	H	nd	> 10	> 10	> 10	> 10	**0.38**	**0.033**	2.2	**0.3**	**0.32**	1.8	2.3	> 10	> 10
**12d**	H	H	CH_3_	H	H	> 10	> 10	> 10	> 10	> 10	> 10	> 10	> 10	> 10	> 10	> 10	> 10	> 10	> 10
**15a**	H	H	H	CH_3_	H	> 10	> 10	> 10	> 10	> 10	**0.19**	1.1	> 10	**0.16**	> 10	> 10	> 10	> 10	> 10
**15b**	H	H	H	C_2_H_5_	H	> 10	> 10	> 10	> 10	> 10	**0.38**	nd	nd	nd	3.9	nd	nd	nd	> 10
**15c**	H	H	H	CH_2_CONH_2_	H	> 10	> 10	> 10	> 10	> 10	> 10	> 10	> 10	> 10	> 10	> 10	> 10	> 10	> 10
**15d**	H	H	H	CH_2_COOC_2_H_5_	H	> 10	> 10	> 10	> 10	> 10	> 10	nd	nd	nd	> 10	nd	nd	nd	> 10
**15e**	H	H	CH_3_	CH_3_	H	> 10	> 10	> 10	> 10	> 10	> 10	> 10	> 10	> 10	> 10	> 10	> 10	> 10	> 10
**16**	H	H	H	H	Br	> 10	> 10	> 10	> 10	> 10	**0.11**	**0.11**	2.8	**0.029**	1.9	1.3	1.8	3.9	> 10
**17a**	H	Ph	H	H	H	> 10	> 10	> 10	> 10	> 10	8.8	7.2	> 10	1.3	> 10	> 10	> 10	> 10	> 10
**17b**	H	2-F-Ph	H	H	H	> 10	> 10	> 10	> 10	> 10	5.8	4.1	> 10	**0.91**	> 10	> 10	> 10	> 10	> 10
**17c**	H	3-F-Ph	H	H	H	> 10	> 10	> 10	> 10	> 10	7.2	> 10	> 10	3.2	> 10	> 10	> 10	> 10	> 10

^a^ IC_50_ values below 1 μM are highlighted in bold. All data points for construction of dose response curves were recorded in triplicate. Typically, the standard deviation of single data points was below 10%. nd: not determined.

## Crystal structure analysis

**Fig 6:**
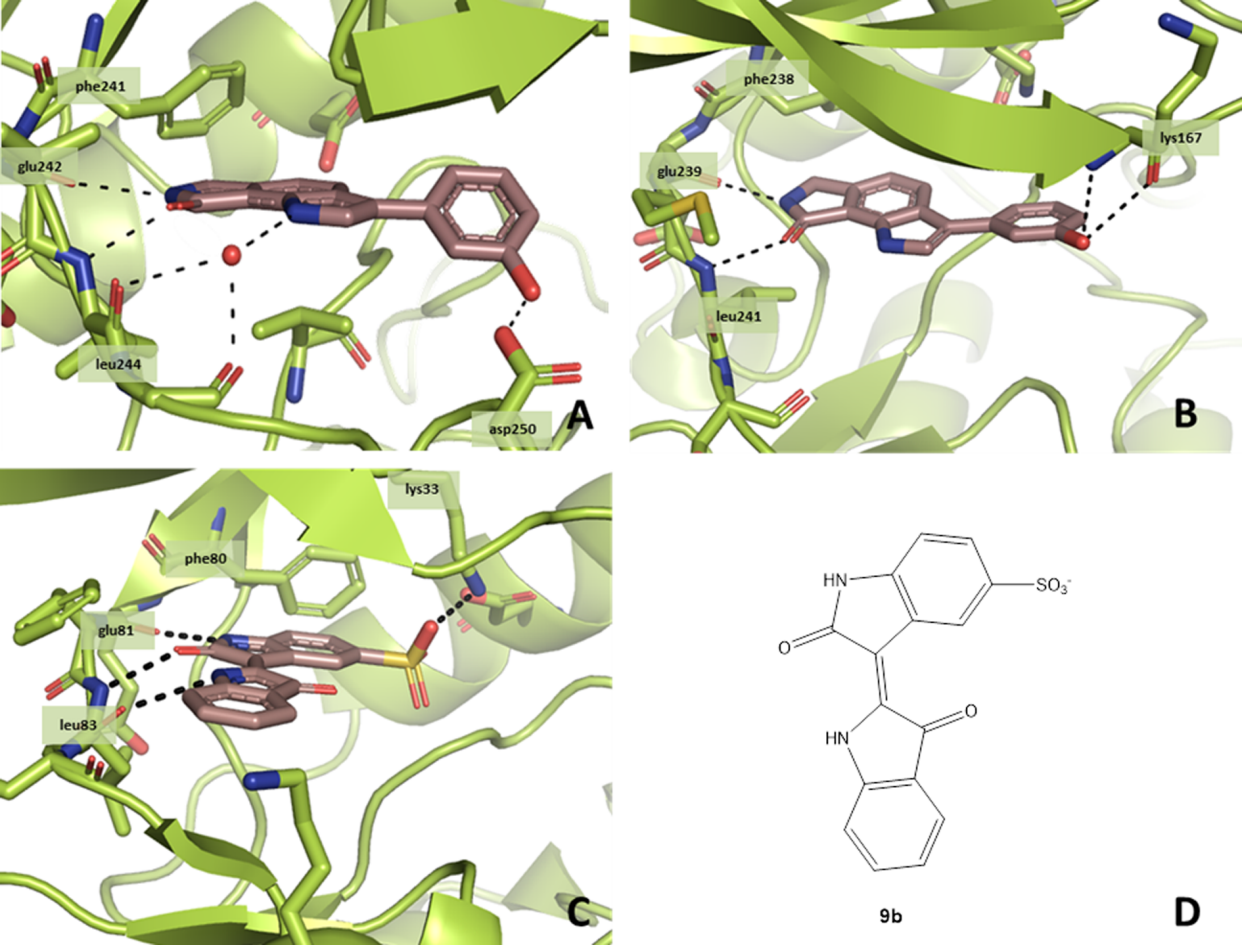
Crystal structures of inhibitor-kinase complexes. **A:** Co-crystal structure of **8g** in CLK1 (PDB-ID: 6FT8). **B:** Docking pose of **8g** in DYRK1A (PDB-ID: 3ANQ [44]). **C:** Co-crystal structure of indirubin-5-sulphonate (**9b**) in CDK2 phosphorylated at Thr160 (PDB-ID: 1E9H [43]). **D:** Indirubin-5-sulphonate **9b**.

To enable experimental analyses of the molecular interaction between 3-aryl-6,7-dihydropyrrolo[3,4-*g*]indol-8(1*H*)-ones and their target enzymes, co-crystallization experiments with some representative congeners and CLK1 or CLK3 were carried out, respectively. From these approaches, **8g/**CLK1, **12a**/CLK1 and **8a**/CLK3 were generated as crystalline inhibitor/protein complexes. The X-ray crystal structure analyses corroborated the general binding mode predicted by the docking studies. For instance, in the complex of CLK1 with the hydroxy compound **8g** (PDB-ID: 6FT8), the ligand forms two hydrogen bonds to the hinge region (Glu242, Leu244). The predicted additional H-bond to Asp250 is formed via the hydroxyl group of the inhibitor. Furthermore, the indole nitrogen indirectly establishes H-bonds to the hinge region through a water molecule which interacts with the carbonyl oxygens of Leu244 and Gly245 (Fig 6A). A comparison of the **8g**/CLK1 crystal structure (Fig 6A) with indirubin-5-sulphonate (**9b**, Fig 6D) cocrystallized with CDK2 (Fig 6C) [43] demonstrates similar binding modes. Both ligands contain pyrrolinone scaffolds which are involved via the nitrogen NH and the carbonyl oxygen in hydrogen bonds to the hinge region amino acids gk+1 and gk+3. In contrast to the pyrrole-annulated isoindolinone **8g**, indirubins such as **9b** consist of two indolinone elements connected by a double bond. Nevertheless, in both crystal structures one of the two NH groups is oriented towards the leucine of the hinge region (gk+3). However, only the indirubin derivative is positioned close enough to establish a direct H-bond to the gk+3 residue (Leu83 in the case of CDK2), whereas for the indole nitrogen of **8g** indirect bonding via a water molecule to the corresponding Leu244 of CLK1 is observed. The sulphonate substituent at position 5 of the indirubin derivative **9b** is involved in a further hydrogen bond to the conserved lysine of CDK2, increasing its potency compared to indirubin (IC_50_ CDK2 **9b**: 35 nM) [43]. In contrast, **8g** is anchored within the ATP-pocket by an additional H-bond between the hydroxyl group and Asp250. As the DYRK kinases are structurally closely related to the CLK family, assessment of inhibitor selectivity against DYRKs is relevant. Fig 6B shows the potential binding mode of **8g** in DYRK1A (PDB-ID: 3ANQ) [44] resulting from a docking analysis. The general orientation is very similar to the one observed in the CLK1 cocrystal structure. Between the lactam function of the inhibitor and the gk+1 and gk+3 residues of the protein two hydrogen bonds are formed, respectively. An increase in the distance of the indole nitrogen to the hinge region, caused by a horizontal shift of the ligand, prevents the formation of a direct H-bond as observed for indirubin. Moreover, due to this shift the position of the phenol at the entrance of the ATP-binding site is changed as well. Instead of hydrogen bonding between the hydroxyl group and Asp247, an interaction with the backbone amide of Lys167 is predicted.

**Fig 7:**
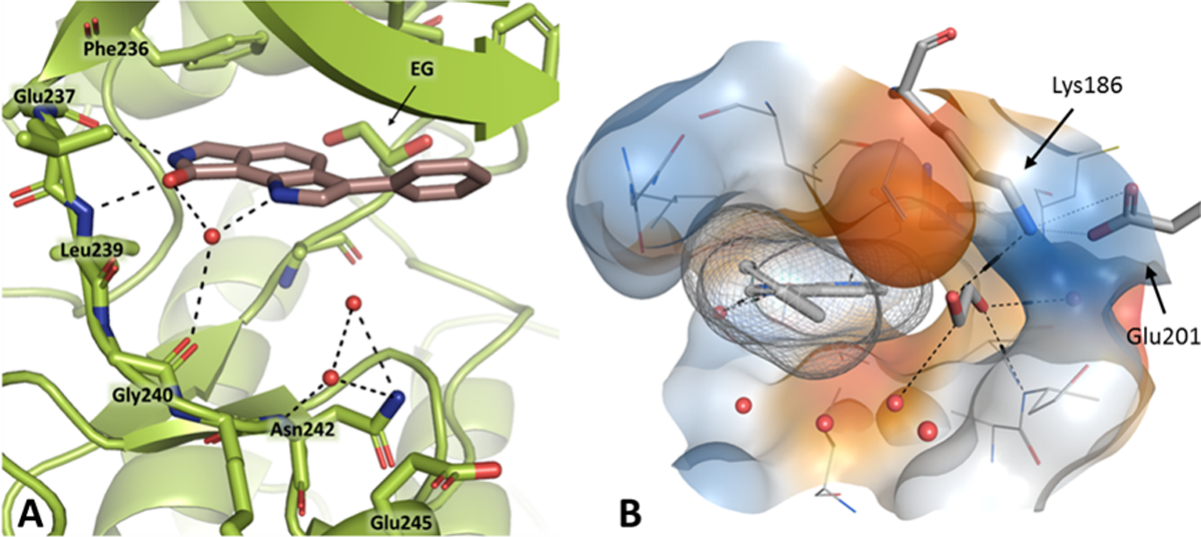
Cocrystal structure of 8a with CLK3 (PDB-ID: 6FT7); red spheres: water molecules; EG: Ethylene glycol; black dashed lines: hydrogen bonds. A: The lactam motif of the ligand forms the canonical hydrogen bonds to the amino acids gk+1 (Glu237) and gk+3 (Leu239); the indole nitrogen is connected to the amino acid gk+4 (Gly240). B: Side view. An ethylene glycol molecule fills a space at the back of the pocket near the αC-helix which is left unoccupied by **8a**.

Although the hit structure **8a** from the initial screening failed to inhibit CLK3 at concentrations below 10 μM, we were able to crystallize this protein kinase in complex with the ligand (PDB-ID: 6FT7). The X-ray structure analysis revealed that the inhibitor is localized in the ATP binding pocket, occupying a position similar to the binding mode predicted by the docking study for the pose in CLK1. As observed in the **8g**/CLK1 complex, the lactam element of **8a** establishes hydrogen bonds to the gk+1 and the gk+3 amino acids of CLK3, and the indole nitrogen forms a water-mediated hydrogen-bond to the hinge region (Fig 7). Compared to the situation of **8g** in CLK1, **8a** is slightly rotated in the binding pocket of CLK3 so that the indole nitrogen is veered away from the hinge region and the phenyl substituent is shifted slightly towards the DFG motif. The cavity near the αC-helix and the DFG motif is not occupied by the ligand, but by an ethylene glycol molecule which is kept in place by hydrogen bonds to the canonical Lys186 and Asp320 of the DFG motif. In further inhibitor design approaches this region of the binding pocket could be addressed by tailored side chains sprouting from the core element of new ligands.

**Fig 8:**
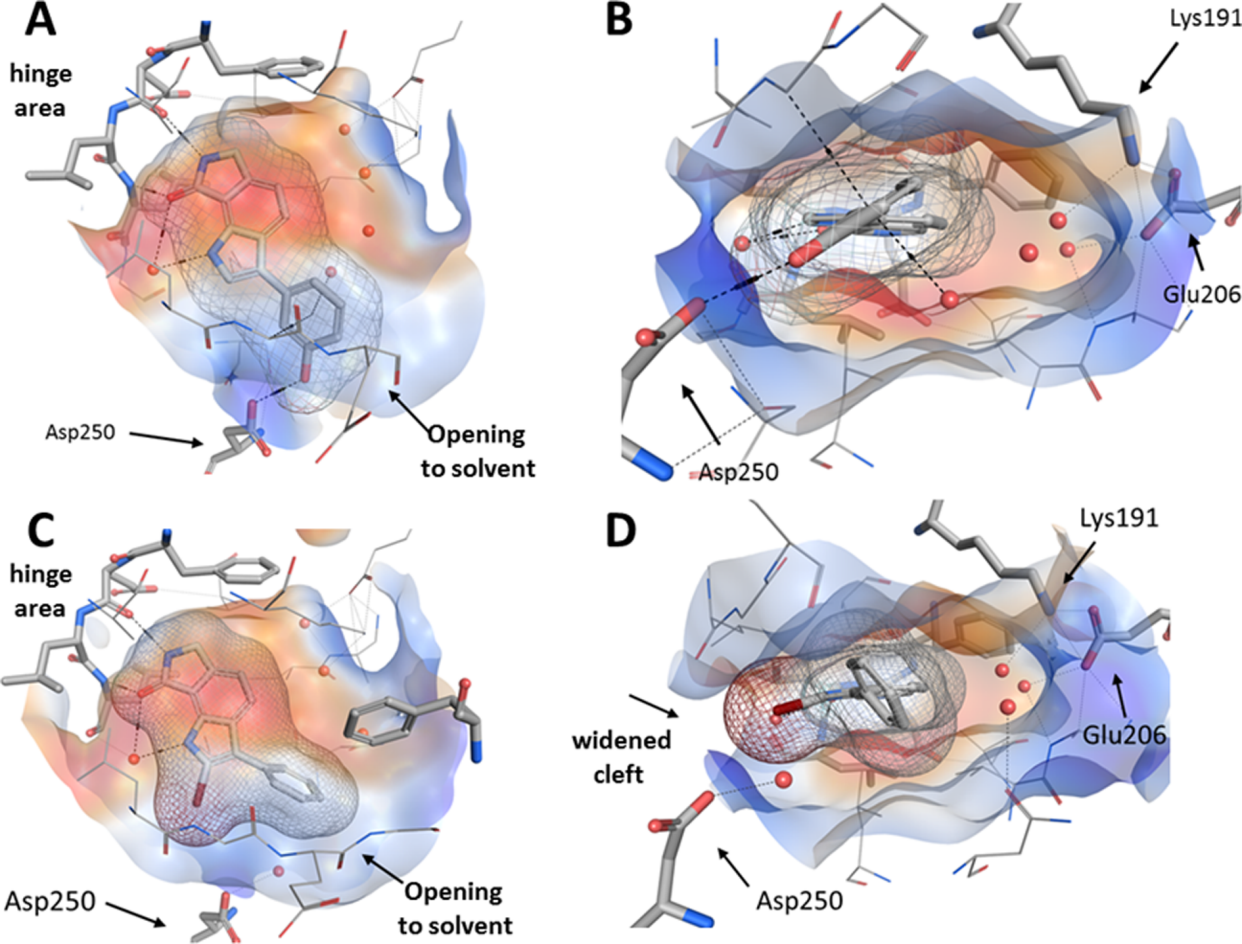
Comparison of co-crystal structures of 8g (PDB-ID: 6FT8; upper row) and 16 (PDB-ID: 6FT9; lower row) in complex with CLK1, respectively; red spheres: water molecules; black dashed lines: hydrogen bonds. A: Top view of **8g.** B: Side view of **8g**. C: Top view of **16**. D: Side view of **16**. A, C: While the lactam motives of the ligands perform the canonical hydrogen bonds to the hinge region, the indole nitrogen atoms are connected to a conserved water molecule. B, D: An area near the Lys191 and Glu206 side chains, unoccupied by both **7g** and **16**, is filled by three water molecules. The opening to the entrance of the binding pocket is delimited by Asp250. Compared to the shape of the pocket with bound **8g** (B), the entrance of the binding pocket is widened to accommodate the 2-bromo substituent of **16** (D).

A third crystallized inhibitor/ligand complex was generated from the 2-bromo-substituted analogue **16** with CLK1 (PDB-ID: 6FT9). The structure analysis revealed a binding mode resembling the orientations of **8g** in CLK1 and of **8a** in CLK3. While the lactam substructure of **16** forms hydrogen bonds to the hinge region, the indole nitrogen is connected to the hinge via hydrogen bonds through a water molecule. The planes of the phenyl substituents in **8g** and **16** are nearly perpendicularly oriented in the two structures. In both complexes, three water molecules occupy the area near the Lys191 and Glu206 side chains. For accommodation of the bromo substituent of **16**, the entrance to the ATP pocket is somewhat widened compared to the situation found with **8g** (Fig 8).

Although by means of selectivity congener **8c** appears to be the most interesting representative of the series, a crystallized complex with CLK1 or one of the other protein kinases mentioned above was not generated up to now. S1 Fig in the supporting information illustrates the putative binding mode of this molecule in CLK1, resulting from a docking experiment.

## Biological evaluation and discussion

The synthesized derivatives of **8a** were evaluated in a panel of CMGC kinases as summarized in Table 1. **8j**, containing a 3-nitro group at the phenyl substituent, was the most potent CLK1 inhibitor in the series **8**. Similar other derivatives with small substituents at the phenyl rings exhibited activity in the same order of magnitude. However, the substitution pattern on the 3-phenyl ring influenced the CLK/DYRK selectivity profile. In case of the 3-hydroxy derivative **8g**, the changes may be attributable to the additional hydrogen bond formed between the phenol OH-group and Asp250 (Fig 6A). Low selectivity as well as reduced activity was observed for the larger acetyloxy substituent in **8h**. Compared to the unsubstituted **8a**, introduction of chloro substituents in 3- or 4-position at the phenyl ring caused a loss of activity but improved CLK1 versus DYRK selectivity (**8c-e**). In contrast, a 2-Cl substituent (**8b**) proved to be disadvantageous in this respect. The overall observation that in series **8** nature and position of the substituents at the 3-phenyl ring produced only a minor effect on CLK1 inhibitory activity corresponds well to its position at the opening of the ATP-binding site. Methylation of the indole nitrogen atom (**15a**) led to a minor loss of CLK1 inhibitory activity whereas methylation at the lactam nitrogen (**12d**) resulted in complete inactivity, underlining the importance of the hydrogen donor ability of this position for binding affinity. The size of the substituent at the indole nitrogen (R^4^ in Table 1) is restricted, because activity is significantly reduced in the presence of an ethyl substituent (**15b**) and no longer detected upon acetamide substitution (**15c**) or the larger acetic acid ethyl ester (**15d**). Regarding substitution at position 5 at the heterocyclic core the IC_50_-values were consistent with the predictions from docking studies. The bromo-substituent of **12a** occupies previously unused space in the back pocket. This feature leads to retained activity on CLK1 (IC_50_ CLK1: 43 nM) on the one hand, but diminished selectivity towards DYRK1A and other tested kinases on the other hand. While the additional 2-methyl substituent in **12b** maintained selectivity towards CDK1, CDK5 and CK1, this congener is still a potent inhibitor of DYRK1A. The observation that the 5-phenyl substituents present in the derivatives **17a-c** are not tolerated without loss of CLK inhibitory activity is well explained by the lack of space in the back pocket for accommodation of these large residues. Substitution of the parent structure **8a** at position 2 by either methyl (**8k**) or bromine (**16**) decreased the selectivity towards the DYRK family more than tenfold. A further loss of selectivity towards CK1 was observed with the larger ethyl substituent (**8l**), although the CLK1 inhibitory activity was not significantly deteriorated. The selectivity profile of the compound class within the CLK family revealed a parallel change of IC_50_ values for CLK1 and CLK4 upon substitution pattern variation at the heterocyclic core scaffold of **8a**. With the exception of **12c**, inhibitory activity on CLK2 was found to be in the same order of magnitude, but consistently lower than on CLK1 and CLK4. In contrast, all congeners showed considerably weaker or lacking inhibition of CLK3. Overall, the biological data shows that it is possible to avoid inhibition of DYRK kinases and create entirely CLK selective molecules, although with the disadvantage of losing potency. In this regard compound **8c** appears to display an optimal balance, exhibiting double digit nanomolar inhibition of CLK1/4 and inactivity on DYRKs up to 10 μM. Selectivity towards the more distantly related CDKs, CK1 and GSK3 is achievable even without such a trade-off. Although the inhibitor/CLK complex crystal structure analyses nicely reproduced the binding modes predicted by the docking experiments, a straightforward explanation for the CLK selectivity observed with **8c** versus DYRK1A is not yet obvious. Perhaps in the smaller ATP binding pocket of CLK1 more hydrophobic contacts to the inhibitor can be established than in the larger pocket of DYRK1A. However, this explanation is at the moment highly speculative and not yet proven by X-ray crystal structure analyses results.

Being the most promising compound in terms of CLK1 inhibitory potency and selectivity, KuWal151 (**8c**) was tested in the National Cancer Institute cancer cell line screening on 57 tumor cell lines of different tissue origin [45]. With only a few exceptions, KuWal151 inhibited the growth of most cell lines in submicromolar concentrations. In Table 2 the cancer cell line inhibition data is listed with the expression rates of SPF45 and CLK1 as indicated in the Expression Atlas database (**https://www.ebi.ac.uk/gxa/home**). Apparently the cell lines with high levels of CLK1 encoding RNA are inhibited over average. The cell line MDA-MB-435 which was most strongly inhibited in the screening panel (GI_**50**_ = 72 nM) expresses a comparably high CLK1-RNA level of 22 FPKM (for an explanation of FPKM, refer to [46]). In contrast, the least sensitive cell line UACC 257, on which **8c** did not reach the GI_**50**_ up to 50 μM, has a comparably low CLK1-RNA level of 12 FPKM. However, the available data are not sufficient to construe a clear relationship between the expression levels of the putative target CLK1 and sensitivity of the cell lines against **8c**. The same holds true for SPF45, an important substrate of CLK1 involved in splicing which is found to be overexpressed in many cancer cell lines. While several of the cell lines with high SPF45 expression levels (HCT-116, HCT-15, HT-29, KM12, MCF-7) are strongly inhibited by **8c**, other (OVCAR-8, HOP-92, MDA-MB-431) were comparably less sensitive to the inhibitor. The lack of a straight relationship between CLK1 and/or SPF45 expression levels on the one hand and sensitivity against the CLK1 inhibitor **8c** on the other hand may have various reasons, e.g. different uptake rates of the inhibitor by the cells or alternative intracellular targets. For verification of the results from the NCI screening, which is based on the in situ fixation of cells followed by staining with the protein-binding dye sulforhodamin B [47,48], **8c** was also tested on two cell lines of the NCI panel (HT-29 and MDA-MB-231) in a crystal violet assay. The results (S2 Table) showed similar inhibition concentrations with GI_**50**_ values in the submicromolar range. In this test system, the human adult primary kidney cell line RC-124 was inhibited by comparable concentrations, indicating that splicing inhibition by the CLK inhibitor KuWal151 (**8c**) affects non-cancerous cells as well.

**Table 2:** In vitro growth inhibition of cancer cell lines by KuWal151 (8c)^a^.

Cell line	Cancer origin	GI50 [μM]	SPF45^b^ [FPKM]^c^	CLK1 [FPKM]^c^
786–0	Renal Cancer	0.275	18	13
A498	Renal Cancer	0.166	10	9
A549/ATCC	Non-Small Cell Lung Cancer	0.389	16	11
ACHN	Renal Cancer	0.372	15	6
BT-549	Breast Cancer	0.204	n.a.	n.a.
CCRF-CEM	Leukemia	0.178	n.a.	n.a.
COLO 205	Colon Cancer	0.209	n.a.	n.a.
DU-145	Prostate Cancer	0.316	22	7
EKVX	Non-Small Cell Lung Cancer	0.355	19	12
HCC-2998	Colon Cancer	0.912	n.a	n.a
HCT-116	Colon Cancer	0.191	22	9
HCT-15	Colon Cancer	0.178	25	15
HL-60(TB)	Leukemia	0.141	9	14
HOP-92	Non-Small Cell Lung Cancer	5.62	19	7
HS 578T	Breast Cancer	0.204	6	8
HT29	Colon Cancer	0.174	19	15
IGROV1	Ovarian Cancer	0.427	21	11
K-562	Leukemia	0.162	18	9
KM12	Colon Cancer	0.209	20	39
LOX IMVI	Melanoma	0.245	17	17
M14	Melanoma	0.132	n.a	n.a
MCF7	Breast Cancer	0.174	19	11
MDA-MB-231/ATCC	Breast Cancer	1.38	18	14
MDA-MB-435	Melanoma	0.0724	11	22
MDA-MB-468	Breast Cancer	0.234	20	11
MOLT-4	Leukemia	0.257	n.a	n.a
NCI/ADR-RES	Ovarian Cancer	0.178	n.a	n.a
NCI-H226	Non-Small Cell Lung Cancer	28.8	8	6
NCI-H23	Non-Small Cell Lung Cancer	0.525	25	12
NCI-H322M	Non-Small Cell Lung Cancer	2.39	7	13
NCI-H460	Non-Small Cell Lung Cancer	0.209	15	4
NCI-H522	Non-Small Cell Lung Cancer	0.158	9	15
OVCAR-3	Ovarian Cancer	0.191	12	11
OVCAR-4	Ovarian Cancer	0.912	15	9
OVCAR-5	Ovarian Cancer	1.25	n.a	n.a
OVCAR-8	Ovarian Cancer	0.724	36	10
PC-3	Prostate Cancer	0.339	12	15
RPMI-8226	Leukemia	0.288	19	10
RXF 393	Renal Cancer	0.457	n.a	n.a
SF-268	CNS Cancer	0.437	12	5
SF-295	CNS Cancer	0.224	11	8
SF-539	CNS Cancer	0.166	12	12
SK-MEL-2	Melanoma	0.316	n.a	n.a
SK-MEL-28	Melanoma	0.692	8	12
SK-MEL-5	Melanoma	0.407	9	9
SK-OV-3	Ovarian Cancer	0.275	20	9
SN12C	Renal Cancer	0.331	n.a	n.a
SNB-19	CNS Cancer	0.389	n.a	n.a
SNB-75	CNS Cancer	0.151	10	11
SR	Leukemia	0.151	n.a	n.a
SW-620	Colon Cancer	0.209	22	17
T-47D	Breast Cancer	0.309	20	7
TK-10	Renal Cancer	10.2	n.a	n.a
U251	CNS Cancer	0.263	n.a	n.a
UACC-257	Melanoma	>50.1	8	12
UACC-62	Melanoma	0.275	10	12
UO-31	Renal Cancer	0.832	10	6

^a^ n.a. = not available

^b^ synonym in Expression Atlas: RBM17

^c^ FPKM (fragments per kilobase of exon model per million reads mapped) is a common unit of gene expression calculated from RNA-sequencing data. The calculation takes into account gene length and total number of mapped reads [46].

## Conclusion

3-Aryl-substituted 6,7-dihydropyrrolo[3,4-*g*]indol-8(1*H*)-ones were identified as inhibitors of the CLK protein kinase family. Among the members of this new inhibitor class, KuWal151 (**8c**) was identified as a CLK1, CLK2 and CLK4 inhibitor with double-digit nanomolar potency and selectivity versus the closely related protein kinases of the DYRK family. The putative orientation of these inhibitors in the ATP binding pocket of CLK1 proposed by docking studies was verified by analysis of three crystal structures of closely related analogues of KuWal151 in complex with CLK1 or CLK3, respectively. In agreement with the binding mode obtained from the crystal structures and based on the biological data, qualitative structure activity relationships were established. In submicromolar concentrations **8c** displayed a strong antiproliferative activity on most cell lines of the NCI cancer cell line screening panel. Thus, KuWal151 (**8c**) might be a suitable starting structure for the development of anticancer agents acting on the splicing machinery of cancer cells.

## Experimental

### Molecular docking

Prior to the molecular docking experiments the protein crystal structure of CLK1 (PDB-ID: 1Z57) [36] was protonated and stripped of water molecules as well as of the ligand DBQ [49]. All new ligand structures were created and energy minimized in MOE (Molecular Operating Environment, 2013.08, Chemical Computing Group Inc., Montreal, Canada). GOLD (version 5.2.2.) [35] was used for the docking experiments. The binding site was defined as a sphere around the cocrystallized ligand DBQ with a radius of 10 Å thus encompassing the ATP-binding pocket. As a scoring function chemscore [50] was applied with kinase specific parameters supplied by GOLD. No constraints were added to the setup. Docking accuracy was set to 200% and early termination was not allowed. The resulting poses were evaluated according to their score as well as by visual inspection of the respective binding mode. Visualization was carried out using PyMol, vers. V0.99 [51] (illustrations with green cartoons of host proteins) or using MOE (Molecular Operating Environment, 2013.08, Chemical Computing Group Inc., Montreal, Canada) (illustrations with brown-and-blue surfaces of host proteins).

### Crystallography and structure analysis

CLK1 and CLK3 have been purified as described [36,52]. Crystallization was performed using sitting-drop vapor diffusion method at 4°C and various conditions as summarized in Table 3. Diffraction data were collected at Diamond Light Source and were processed and scaled with Mosflm [53] and Scala [54], respectively. Structures were solved by molecular replacement method using Phaser [55] and the coordinates of published CLK1 and CLK3 structure [36,52]. Model rebuilding was performed in COOT [56] and the structures were refined using REFMAC [57]. Geometric correctness was verified using MOLPROBITY [58]. The data collection and refinement statistics are summarized in Table 3.

**Table 3:** Data collection and refinement statistics of CLK/ligand complexes.

Complex	CLK1-8g	CLK1-16	CLK3-8a
accession code	6FT8	6FT9	6FT7
***Data Collection***			
Beamline	Diamond, I04	Diamond, I03	Diamond, I02
Wavelength (Å)	0.97949	0.97624	0.97950
Resolution^a^ (Å)	23.65–1.45 (1.53–1.45)	29.11–1.87 (1.97–1.87)	59.94–2.02 (2.13–2.02)
Spacegroup	*C*2	*P*2_1_	*P*2_1_
Cell dimensions	*a* = 91.6, *b =* 64.2, *c* = 88.4 Å	*a* = 56.4, *b =* 116.3, *c* = 91.3 Å	*a* = 61.8, *b =* 116.8, *c* = 69.9 Å
	*α* = *γ* = 90.0˚, *β* = 127.7˚	*α* = *γ* = 90.0˚, *β* = 99.0˚	*α* = *γ* = 90.0˚, *β* = 92.8˚
No. unique reflections^a^	71,117 (10,186)	95,883 (13,962)	62,659 (9,103)
Completeness^a^ (%)	99.1 (97.5)	100.0 (100.0)	96.7 (96.0)
I/σI^a^	9.9 (2.2)	9.9 (2.0)	8.0 (2.1)
R_merge_^a^ (%)	0.044 (0.381)	0.083 (0.738)	0.091 (0.668)
Redundancy^a^	3.0 (2.9)	5.2 (5.0)	3.7 (3.6)
***Refinement***			
No. atoms in refinement (P/L/O)^b^	2,814/40/484	8,297/120/602	5,896/38/547
B factor (P/L/O)^b^ (Å^2^)	25/18/38	42/29/46	43/39/45
R_fact_ (%)	16.4	16.6	19.2
R_free_ (%)	19.5	20.2	23.5
rms deviation bond^c^ (Å)	0.016	0.016	0.015
rms deviation angle^c^ (°)	1.7	1.6	1.6
***Molprobity Ramachandran***			
Favour (%)	97.92	97.91	96.05
Outlier (%)	0	0	0
Crystallization conditions	20% PEG3350, 0.1 M sodium malonate	20% 1,2-propanediol, 10% glycerol, 0.1 M sodium/potassium phosphate	21% PEG3350, 0.2 M sodium iodide, 0.1 M bis-tris-propane pH 7.0, 10% ethylene glycol

^a^ Values in brackets show the statistics for the highest resolution shells.

^b^ P/L/O indicate protein, ligand molecules presented in the active sites, and other (water and solvent molecules), respectively.

^c^ rms indicates root-mean-square.

### Protein kinase assays [26]

Buffers. Buffer A: 10 mM MgCl_2_, 1 mM EGTA, 1 mM DTT, 25 mM Tris-HCl pH 7.5, 50 μg heparin/mL. Buffer C: 60 mM β-glycerophosphate, 15 mM p-nitrophenylphosphate, 25 mM MOPS (pH 7.2), 15 mM EGTA, 15 mM MgCl_2_, 1 mM DTT, 1 mM sodium vanadate, 1 mM phenylphosphate.

Kinase activities were assayed in buffer A or C at 30°C at a final ATP concentration of 15 μmol/L. Blank values were subtracted and activities were expressed in percent of the maximal activity, i.e. in the absence of inhibitors. Controls were performed with appropriate dilutions of DMSO. The GS-1, CKS, CDK7/9 tide and RS peptide substrates were obtained from Proteogenix (Oberhausbergen, France).

CDK1/cyclin B (M phase starfish oocytes, native), CDK2/cyclin A and CDK5/p25 (human, recombinant) were prepared as previously described [33]. Their kinase activity was assayed in buffer A, with 1 mg histone H1/mL, in the presence of 15 μmol/L [γ-33P] ATP (3,000 Ci/mmol; 10 mCi/mL) in a final volume of 30 μL. After 30 min incubation at 30°C, the reaction was stopped by harvesting onto P81 phosphocellulose supernatant (Whatman) using a FilterMate harvester (Packard) and washing in 1% phosphoric acid. Scintillation fluid was added and the radioactivity measured in a Packard counter.

CDK9/cyclin T (human, recombinant, expressed in insect cells) was assayed as described for CDK1/cyclin B, but using CDK7/9 tide (YSPTSPSYSPTSPSYSPTSPSKKKK) (8.1 μg/assay) as a substrate.

GSK-3 (porcine brain, native, affinity purified on axin1-sepharose beads) was assayed, as described for CDK1 with 0.5 mg BSA /mL + 1 mM DTT and using a GSK-3 specific substrate (GS-1: YRRAAVPPSPSLSRHSSPHQSpEDEEE) (pS stands for phosphorylated serine) [59].

CK1δ/ε (porcine brain, native, affinity purified on axin2-sepharose beads) was assayed as described for CDK1 but in buffer C and using 25 μM CKS peptide (RRKHAAIGpSAYSITA), a CK1-specific substrate[60].

CLK1, 2, 3 and 4 (mouse, recombinant, expressed in *E*. *coli* as GST fusion proteins) were assayed as described for CDK1/cyclin B with 0.5 mg BSA /mL + 1 mM DTT and RS peptide (GRSRSRSRSRSR) (1μg/assay) as a substrate.

DYRK1A, 1B, 2, 3 (human, recombinant, expressed in *E*. *coli* as GST fusion proteins), CLK1, 2, 3, and 4 (mouse, recombinant, expressed in *E*. *coli* as GST fusion proteins) were assayed in Buffer A (supplemented extemporaneously with 0.15 mg BSA/mL + 1 mM DTT) with 1 μg of RS peptide (GRSRSRSRSRSR) as a substrate.

All data points for construction of dose response curves were recorded in triplicate. Typically, the standard deviation of single data points was below 10%.

### Cell culture

HT-29 colon carcinoma cells, MDA-MB-231 breast cancer cells, MCF-7 breast carcinoma cells (all supplied by Leibniz Institute DSMZ—German Collection of Microorganisms and Cell Cultures, Braunschweig, Germany) were maintained in Dulbecco's Modified Eagle Medium (4.5 g/L D-glucose, L-glutamine, pyruvate), which was supplemented with gentamycin (50 mg/L) and fetal bovine serum superior, standardized (Biochrom GmbH, Berlin) (10% v/v), and were passaged once a week. RC-124 healthy human kidney cells (supplied by CLS Cell Lines Service GmbH, Eppelheim, Germany) were maintained in McCoy's 5A (modified, with L-glutamine) medium which was supplemented with gentamycin (50 mg/L) and fetal bovine serum superior, standardized (Biochrom GmbH, Berlin) (10% v/v), and were also passaged once a week. For experiments with RC-124 cells, microtiter plates had been pretreated in the following way: 30 μL of a sterilized gelatine solution (1.5% (m/V)) were added to each well of flat bottom 96-well plates, the plates were covered with their lids, incubated for 1h at 37°C, the excess solution was removed, the wells were washed with PBS 7.4 pH, and the new cell-culture medium was added.

### Crystal violet proliferation assay

The antiproliferative effects were determined according to a routinely used method. In short: a volume of 100 μL of HT-29 cells (2565 cells/mL), MDA-MB-231 cells (4120 cells/mL), MCF-7 cells (4840 cells/mL) or RC-124 cells (1460 cells/mL) was transferred into the wells of 96-well plates (note: for RC-124 pretreated plates were used, see above) and incubated at 37°C/5% CO_2_ for 72 h (MCF-7, MDA-MB-231, RC-124) or 48 h (HT-29). Stock solutions of **8c** in DMSO were prepared and diluted with the respective cell culture medium to graded concentrations (final concentration of DMF: 0.1% v/v). After 72 h (HT-29) or 96 h (MCF-7, MDA-MB-231, RC-124) of exposure, the cell biomass was determined by crystal violet staining and the IC_50_ value was determined as the concentration that caused 50% inhibition of cell proliferation compared to an untreated control. Results were calculated as the mean values of three independent experiments.

### Synthesis of test compounds

#### General

In general, starting materials were purchased from Acros Organics (Geel, Belgium) or Sigma-Aldrich (Steinheim, Germany). 7-Aminoisoindolin-1-one (**10a**) was purchased from Enamine (Monmouth, U.S.A). Solvents were used as commercially available grades for synthesis, with the exception of THFand CH_2_Cl_2_ which were dried and purified by published methods [61].

Melting points (mp) were determined on an electric variable heater (Electrothermal IA 9100, Bibby Scientific, Stone, United Kingdom) in open glass capillaries. IR-spectra were recorded as KBr discs on a Thermo Nicolet FT-IR 200 (Thermo Nicolet, Madison, WI, USA). ^1^H-NMR-spectra and ^13^C-NMR-spectra were recorded on the following instruments: Bruker Avance DRX-400, Bruker Avance III-400 and Bruker Avance II-600 (Bruker Corporation, Billerica, MA, USA); internal standard tetramethylsilane; signals in ppm (δ scale). Signals in ^13^C spectra were assigned based on the results of ^13^C DEPT135 experiments (NMR Laboratories of the Chemical Institutes of the Technische Universität Braunschweig). Elemental analyses were determined on a CE Instruments FlashEA 1112 Elemental Analyser (Thermo Quest) (Thermo Quest, San Jose, CA, USA). Mass spectra were recorded on a Finnigan-MAT 95 (Thermo Finnigan MAT, Bremen, Germany). Accurate measurements were conducted according to the peakmatch method using perfluorokerosene (PFK) as an internal mass reference; (EI)-MS: ionisation energy 70 eV (Department of Mass Spectrometry of the Chemical Institutes of the Technische Universität Braunschweig). TLC: Polygram Sil G/UV_254_, 40 x 80 mm, (Macherey-Nagel, Düren, Germany), visualization by UV-illumination (254 nm and 366 nm). Purity was determined by HPLC using the following devices and settings: isocratic elution: Elite LaChrom (Merck/Hitachi), Pump L-2130, autosampler L-2200, Diode Array Detector L-2450, organizer box L-2000; column: Merck LiChroCART 125–4, LiChrosphere 100, RP 18, 5 μm; flow rate 1.000 mL/min, volume of injection: 10 μL; detection (DAD) at 254 and 280 nm; AUC-%-method.; time of detection 15 min, net retention time (t_*N*_), dead time (t_m_) related to DMSO; gradient elution: Elite LaChrom (Merck, Darmstadt, Germany), Pump L-2130, autosampler L-2200, UV Detector L-2400, organizer box L-2000; column: Merck LiChroCART 125–4, LiChrosphere 100, RP 18, 5 μm; flow rate 1.000 mL/min, volume of injection: 10 μL; detection at 254 nm; AUC-%-method.; net retention time (t_*N*_), dead time (t_m_) related to DMSO. For all gradient runs, mixtures of ACN and water were used as specified for particular compounds. Preparation of H_2_O+(Et_3_NH)_2_SO_4_-buffer (*p*H 2.6) for isocratic HPLC: triethylamine (20.0 mL) and sodium hydroxide (242 mg) are dissolved in water to 1 L. The solution is adjusted to *p*H 2.6 by addition of sulfuric acid. Absorption maxima (λ_max_) were extracted from the spectra recorded by the DAD in the HPLC peak maxima in isocratic runs (software: EZ Chrom Elite Client/server version 3.1.3.). HPLC gradient 1: (acetonitrile/water) 0-10 min (10/90→70/30, linear), 10 min-10.5 min (70/30→90/10, linear), 10.5 min-16.5 min (90/10); HPLC gradient 2: (acetonitrile/water) 0-2 min (10/90), 2 min-12 min (10/90→90/10, linear), 12 min-22 min (90/10).

*7-Amino-4-bromoisoindolin-1-one (****10b****)*. 7-Aminoisoindolin-1-one (**10a**, 148 mg, 1.00 mmol) was suspended in dichloromethane (8 mL) and cooled to -7°C. *N*-Bromosuccinimide (178 mg, 1.00 mmol) was added and the mixture was stirred for 1h. Na_2_S_2_O_3_ (2.3 mL, 10%) was added and stirring continued for another 20 min. The aqueous phase was extracted with CH_2_Cl_2_ (2 x 10 mL), the combined organic phases were washed with water (3 x 10 mL) and brine (10 mL) and dried over Na_2_SO_4_. The solvent was evaporated under vacuum. The raw product was used without further purification.

Light brown crystals, 177 mg (78%). ^1^H-NMR (DMSO-d_6_, 400 MHz): δ 4.14 (s, 1H, CH_2_), 6.20 (s, 2H, amine-NH), 6.55 (d, 1H, *J* = 8.6 Hz), 7.32 (d, 1H, *J* = 8.6 Hz), 8.38 (s, 1H, pyrrolidone-NH); ^13^C NMR (DMSO-d_6_, 101 MHz): δ 45.35 (CH_2_); 115.01, 134.86 (CH); 100.37, 115.75, 144.46, 146.38, 171.53 (C); C_8_H_7_BrN_2_O (227.1).

*7-Amino-4-chloroisoindolin-1-one (****10c****)*. 7-Aminoisoindolin-1-one (**10a**, 296 mg, 2.00 mmol) was dissolved in acetonitrile (20 mL) and heated to 60°C. After the addition of *N*-chlorosuccinimide (294 mg, 2.20 mmol) the mixture was refluxed for 3.5 h. The mixture was then partitioned between ethyl acetate (30 mL) and NaOH solution (5%, 2 x 40 mL). The organic phase was washed with water (40 mL), dried over Na_2_SO_4_ and the solvent was evaporated under vacuum. Further usage as raw product.

Yellow-brown solid, 303 mg (81%). ^1^H NMR (DMSO-d_6_, 400 MHz): δ 4.21 (s, 2H, pyrrolidone-CH_2_), 6.16 (s, 2H, NH_2_), 6.59 (d, 1H, *J* = 8.6 Hz), 7.21 (d, 1H, *J* = 8.6 Hz), 8.38 (s, 1H, pyrrolidone-NH); ^13^C NMR (DMSO-d_6_, 101 MHz): δ 43.75 (CH_2_), 114.54, 132.12 (CH), 112.82, 115.34, 142.25, 145.93, 171.43 (C); C_8_H_7_ClN_2_O (282.6).

*7-Amino-2-methylisoindolin-1-one (****10d****)*. 7-Aminoisoindolin-1-one (**10a**, 74 mg, 0.5 mmol) was dissolved in dry THF (5 mL) under N_2_ atmosphere. At room temperature potassium *tert*-butylat (56 mg, 0.5 mmol) was added and the mixture was stirred for 1 h. Subsequently iodomethane (310 μL, 5.00 mmol) was added and stirring continued for 24 h. After the addition of silica gel (1.5 g) the solvent was evaporated under vacuum. Purification was done by column chromatography (ethyl acetate).

Yellow solid, 44 mg (54.3%), mp.^1^H-NMR (DMSO-d_6_, 400 MHz): δ 2.98 (s, 3H, CH_3_), 4.30 (s, 2H, CH_2_), 6.01 (br s, 2H, amine-NH), 6.54 (dd, 1H, *J* = 0.8 Hz, 8.0 Hz), 6.61 (dd, 1H, *J* = 0.9 Hz, 7.4 Hz), 7.17 (dd, 1H, *J* = 7.3 Hz, 8.1 Hz); ^13^C-NMR (DMSO-d_6_, 101 MHz): δ 29.10 (CH_3_), 52.14 (CH_2_); 113.53, 116.28, 132.68 (CH); 117.61, 142.90, 143.55, 168.15 (C); C_9_H_10_N_2_O (213.7).

#### Synthesis of aryl hydrazinium chlorides 11a-11d

A solution of NaNO_2_ (76 mg, 1.1 mmol) in water (3 mL) was added dropwise to a cooled (< 0°C) suspension of the respective 7-aminoisoindolin-1-one (**10a**, **10d**, 1.00 equiv.; **10b-c**, raw products used as obtained from the synthesis described above) in HCl (37%, 8 mL). After the mixture had cleared it was added to a cooled suspension of SnCl_2_ x 2H_2_O (677 mg, 3.00 mmol) in HCl (37%, 8 mL). The resulting suspension was then stirred at < 0°C for 30 min and afterwards stored at 8°C for 12 h to allow precipitation. The precipitate was isolated by vacuum filtration and washed with petrol ether. Without further purification the hydrazine was used for the following reactions.

*2-(3-Oxoisoindolin-4-yl)hydrazin-1-ium chloride (****11a****)*. From 7-aminoisoindolin-1-one (**10a**, 296 mg, 2.00 mmol), sodium nitrite (166 mg, 2.20 mmol) and tin(II)chloride-dihydrate (1354 mg, 6.00 mmol). Brown powder, 670 mg raw product. ^1^H NMR (DMSO-d_6_, 400 MHz): δ 4.44 (s, 2H, CH_2_), 7.03–7.10 (m, 1H), 7.10–7.17 (m, 1H), 7.46–7.56 (m, 1H), 8.54 (s, 1H, pyrrolinone-NH), 9.03 (s, 1H, NH), 10.48 (br s, 3H, NH_3_^+^); ^13^C NMR (DMSO-d_6_, 101 MHz): δ (ppm) 45.81 (CH_2_); 113.34, 116.45, 132.96 (CH); 117.59, 143.66, 146.13, 171.17 (C); C_8_H_10_ClN_3_OxH_2_O (217.7).

*2-(7-Bromo-3-oxoisoindolin-4-yl)hydrazin-1-ium chloride (****11b****)*. From 7-amino-4-bromoisoindolin-1-one (**10b**, 456 mg, 2.00 mmol), sodium nitrite (166 mg, 2.20 mmol) and tin(II)chloride-dihydrate (1354 mg, 6.00 mmol). Beige solid, 690 mg raw product. ^1^H NMR (DMSO-d_6_, 400 MHz): δ 4.35 (s, 2H, CH_2_), 7.08 (d, 1H, *J* = 8.6), 7.72 (d, 1H, *J* = 8.6), 8.67 (br s, 1H, pyrrolidone-NH), 9.21 (s, 1H, hydrazine-NH), 10.66 (s, 3H, hydrazine-NH_3_^+^); ^13^C NMR (DMSO-d_6_, 101 MHz): δ 46.50 (CH_2_); 115.75, 135.45 (CH); 107.93, 119.38, 143.11, 145.34, 170.34 (C); C_8_H_9_BrClN_3_O (278.5).

*2-(7-Chloro-3-oxoisoindolin-4-yl)hydrazin-1-ium chloride (****11c****)*. From 7-amino-4-chloroisoindolin-1-one (**10c**, 457 mg, 2.50 mmol), sodium nitrite (190 mg, 2.75 mmol) and tin(II)chloride-dihydrate (1692 mg, 7.50 mmol). Beige solid, 640 mg raw product. ^1^H NMR (DMSO-d_6_, 400 MHz): δ 4.39 (s, 2H, pyrrolidone-CH_2_), 7.08 (d, 1H, *J* = 8.7 Hz), 7.55 (d, 1H, *J* = 8.6 Hz), 8.39 (s, 1H, pyrrolidone-NH), 9.04 (s, 1H, hydrazine-NH), 9.95 (br s, 3H, hydrazinium-NH_3_^+^); ^13^C NMR (DMSO-d_6_, 101 MHz): δ 44.71 (CH_2_), 114.38, 132.54 (CH), 118.23, 118.38, 118.61, 142.95, 170.73 (C); C_8_H_9_Cl_2_N_3_O (234.1).

*2-(2-Methyl-3-oxoisoindolin-4-yl)hydrazin-1-ium chloride (****11d****)*. From 7-amino-2-methylisoindolin-1-one (**10d**, 174 mg, 1.07 mmol), sodium nitrite (81 mg, 1.18 mmol) and tin(II)chloride-dihydrate (724 mg, 3.21 mmol). Brown solid, 455 mg raw product. IR (KBr): 3434 cm^-1^ (NH), 3297 cm^-1^ (NH), 1605 cm^-1^ (C = O); 1H NMR (DMSO-d_6_, 400 MHz): δ 3.08 (s, 3H, CH_3_), 7.06 (d, 1H, *J* = 8.1 Hz), 7.16 (d, 1H, *J* = 7.5 Hz), 7.51 (dd, 1H, *J* = 7.5 Hz, 8.2 Hz), 8.5 (br s, 1H, hydrazine-NH), 10.51 (s, 3H, hydrazine-NH); ^13^C NMR (DMSO-d_6_, 101 MHz): δ 29.10 (CH_3_), 52.14 (CH_2_); 113.53, 116.28, 132.68 (CH); 117.61, 142.90, 143.55, 168.15 (C); C_9_H_10_N_2_O (213.7).

#### Synthesis of compounds 8a, 8c, 8d, 8e, 8i, 8k, 12a-d

The corresponding 2-(3-oxoisoindolin-4-yl)hydrazin-1-ium chloride (1.00 equiv) was dispersed in 10 mL acetic acid together with sodium acetate (83 mg, 1.0 equiv) and an appropriately substituted phenylacetaldehyde, or the corresponding dimethyl acetal, or an appropriately substituted ketone (1.00–1.50 equiv). The mixture was heated at 90–100°C for 3.5 h. After cooling and addition of aqueous sodium acetate solution (5%, 20 mL), the mixture was partitioned between water (30 mL) and ethyl acetate (5 x 30 mL). The combined organic layers were dried over Na_2_SO_4_. After addition of silica gel (1.5 g), the solvent was evaporated. Purification by column chromatography (toluene, ethyl acetate, acetic acid) and recrystallization from ethanol.

*3-Phenyl-6*,*7-dihydropyrrolo[3*,*4-g]indol-8(1H)-one (****8a****)*. From phenylacetaldehyde dimethylacetal (665 μL, 4.00 mmol), 2-(3-oxoisoindolin-4-yl)hydrazin-1-ium chloride (**11a**, 1340 mg, raw product obtained as described above) and sodium acetate (328 mg, 4.00 mmol). Yellow solid, mp 294–295°C, 307 mg (31%). IR (KBr): 3435 cm^-1^ (NH), 3200 cm^-1^, 1661 cm^-1^ (C = O); ^1^H NMR (DMSO-d_6_, 400 MHz): δ 4.47 (s, 2H, CH_2_), 7.22–7.32 (m, 2H), 7.40–7.50 (m, 2H), 7.61 (d, 1H, *J* = 2.6 Hz), 7.66–7.75 (m, 2H), 8.07 (d, 1H, *J* = 8.2 Hz), 8.5 (s, 1H, pyrrolidone-NH), 11.8 (s, 1H, indole-NH); ^13^C NMR (DMSO-d_6_, 101 MHz): δ 45.62 (CH_2_), 114.71, 122.78, 123.93, 125.63, 126.85 (2C), 128.82 (2C) (CH), 116.34, 116.59, 125.28, 130.85, 135.26, 139.56, 170.44 (C); C_16_H_12_N_2_O (248.3); MS (EI) *m/z* 248.1 [M]^+^, HRMS (EI): *m/z* [M-H]^+^ calcd 247.08659, obsd 247.08545.

*3-(3-Chlorophenyl)-6*,*7-dihydropyrrolo[3*,*4-g]indol-8(1H)-one (****8c****)*. From 3-chlorophenylacetaldehyde (232 mg, 1.50 mmol), 2-(3-oxoisoindolin-4-yl)hydrazin-1-ium chloride (**11a**, 335 mg, raw product obtained as described above) and sodium acetate (82 mg, 1.0 mmol). Red brown powder, mp 215–216°C, 57 mg (20%); IR (KBr): 3445 cm^-1^, 3423 cm^-1^ und 3362 (NH), 1660 cm^-1^ (C = O); ^1^H NMR (DMSO-d_6_, 400 MHz): δ 4.48 (s, 2H, CH_2_), 7,31 (m, 2H), 7.47 (t, 1H, *J* = 7.9 Hz), 7.71 (m, 3H), 8.07 (d, 1H, *J* = 8.2 Hz), 8.48 (s, 1H, pyrrolinone-NH), 11.91 (s, 1H, indole-NH); ^13^C NMR (DMSO-d_6_, 101 MHz): δ 45.63 (CH_2_); 115.07, 122.58, 124.96, 125.02, 125.33, 126.17, 130.63 (CH); 114.83, 116.72, 124.98, 130.89, 133.54, 137.53, 139.75, 170.33 (C); C_16_H_11_ClN_2_O (282.7); MS (EI) *m/z* 282 [M]^+.^; HRMS (EI) *m/z* [M-H]^+^ calcd 281.04762, obsd 281.04697.

*3-(4-Chlorophenyl)-6*,*7-dihydropyrrolo[3*,*4-g]indol-8(1H)-one (****8d****)*. From 4-chlorophenylacetaldehyde (155 mg, 1.00 mmol), 2-(3-oxoisoindolin-4-yl)hydrazin-1-ium chloride (**11a**, 335 mg, raw product obtained as described above) and sodium acetate (82 mg, 1.0 mmol). White-yellow solid, mp 255–257°C, 59 mg (21%); IR (KBr): 3429 cm^-1^ and 3217 cm^-1^ (NH), 1662 cm^-1^ (C = O); ^1^H NMR (DMSO-d_6_, 400 MHz): δ 4.47 (s, 2H, CH_2_), 7.28 (d, 1H, *J* = 8.2 Hz), 7.48 (d, 2H, *J* = 8.5 Hz), 7.67 (d, 1H), 7.74(d, 2H, *J* = 8.5 Hz), 8.06 (d, 1H, *J* = 8.2 Hz), 8.46 (s, 1H, pyrrolinone-NH), 11.86 (s, 1H, indole-NH); ^13^C NMR (DMSO-d_6_, 101 MHz): δ 45.62 (CH_2_); 114.91, 122.65, 124.46, 128.39, 128.74 (CH); 115.04, 116.67, 125.04, 129.97, 130.88, 134.19, 139.69, 170.36 (C); C_16_H_11_ClN_2_O (282.7); MS (EI) *m/z* 282 [M]^+.^; HRMS (EI) *m/z* [M]^+.^ calcd 282.05544, obsd 282.05486.

*3-(3*,*4-Dichlorophenyl)-6*,*7-dihydropyrrolo[3*,*4-g]indol-8(1H)-one (****8e****)*. From 3,4-dichlorophenyl-acetaldehyde (284 mg, 1.50 mmol), 2-(3-oxoisoindolin-4-yl)hydrazin-1-ium chloride (**11a**, 335 mg, raw product obtained as described above) and sodium acetate (82 mg, 1.0 mmol). White-yellow solid, mp 258–259°C, 30 mg (10%); IR (KBr): 3345 cm^-1^ and 3356 cm^-1^ (NH), 1672 cm^-1^ (C = O); ^1^H NMR (DMSO-d_6_, 400 MHz): δ 4.48 (s, 2H, CH_2_), 7.31 (d, 1H, *J* = 8.3), 7.67 (d, 1H, *J* = 8.4), 7.74 (dd, 1H, *J* = 8.4, 2.1), 7.80 (d, 1H, *J* = 2.7), 7.94 (d, 1H, *J* = 2.1), 8.07 (d, 1H, *J* = 8.3), 8.46 (s, 1H, pyrrolinone-NH), 11.95 (br s, 1H, indole-NH); ^13^C NMR (DMSO-d_6_, 101 MHz): δ (ppm) = 45.62 (CH_2_); 115.19, 122.51, 125.41, 126.73, 128.02, 130.90 (CH); 113.78, 116.76, 124.81, 127.65, 130.81, 131.46, 136.17, 139.82, 170.25 (C); C_16_H_10_Cl_2_N_2_O (317.18); MS (EI): *m/z* 316 [M]^+.^; HRMS (EI): *m/z* [M]^+.^ calcd 316.01647, obsd 316.01636.

*3-(3-Methylphenyl)-6*,*7-dihydropyrrolo[3*,*4-g]indol-8(1H)-one (****8i****)*. From 3-methylphenylacetaldehyde (141 mg, 1.10 mmol), 2-(3-oxoisoindolin-4-yl)hydrazin-1-ium chloride (**11a**, 335 mg, raw product obtained as described above) and sodium acetate (82 mg, 1.0 mmol). Light yellow powder, mp 231–232°C, 36 mg (14%); IR (KBr): 3424 cm^-1^ and 3261 cm^-1^ (NH), 1682 cm^-1^ (C = O); ^1^H NMR (DMSO-d_6_, 600 MHz): δ 2.38 (s, 3H, CH_3_) 4.47 (s, 2H, CH_2_), 7.08 (d, 1H, *J* = 7.4 Hz), 7.27 (d, 1H, *J* = 8.3 Hz), 7.33 (t, 1H, *J* = 7.6), 7.50 (m, 2H), 7.58 (d, 1H, *J* = 2.5 Hz), 8.07 (d, 1H, *J* = 8.3 Hz) 8.45 (s, 1H, pyrrolinone-NH), 11.75 (s, 1H, indole-NH); ^13^C NMR (DMSO-d_6_, 151 MHz): δ 21.16 (CH_3_); 45.62 (CH_2_); 114.64, 122.86, 123.84, 123.98, 126.33, 127.49, 128.70 (CH); 130.83, 135.15, 137.85, 139.51, 170.46 (C); C_17_H_14_N_2_O (262.11); MS (EI): *m/z* 262 [M]^+.^; HRMS (EI): *m/z* [M]^+.^ calcd 262.11006, obsd 262.10972.

*2-Methyl-3-phenyl-6*,*7-dihydropyrrolo[3*,*4-g]indol-8(1H)-one (****8k****)*. From phenylacetone (270 μL, 2.00 mmol), 2-(3-oxoisoindolin-4-yl)hydrazin-1-ium chloride (**11a**, 670 mg, raw product obtained as described above) and sodium acetate (164 mg, 2.0 mmol). Red brown powder, mp 245–247°C, 102 mg (19%); IR (KBr): 3420cm^-1^ (NH), 1684cm^-1^(C = O), 1637cm^-1^; ^1^H NMR (DMSO-d_6_, 400 MHz): 2.50 (s, 3H, CH_3_), 4.44 (s, 2H, CH_2_), 7.16 (d, 1H, *J* = 8.1 Hz), 7.25–7.36 (m, 1H), 7.43–7.53 (m, 4H), 7.72 (d, 1H, *J* = 8.1 Hz), 8.38 (s, 1H, pyrrolidone-NH), 11.57 (s, 1H, indole-NH); ^13^C NMR (DMSO-d_6_, 101 MHz): δ 12.12 (CH_3_); 45.51 (CH_2_); 114.14, 121.37, 125.53, 128.54, 128.89 (CH); 112.84, 115.67, 127.22, 129.38, 133.38, 135.11, 138.50, 170.54 (C); C_17_H_14_N_2_O (262.3); MS (EI) *m/z* 262.1 [M]^+.^, HRMS (EI) *m/z* [M]^+.^ calcd 262.11006, obsd 262.10970.

*2-Ethyl-3-phenyl-6*,*7-dihydropyrrolo[3*,*4-g]indol-8(1H)-one (****8l****)*. From 1-phenylbutan-2-one (300 μL, 2.00 mmol), 2-(3-oxoisoindolin-4-yl)hydrazin-1-ium chloride (**11a**, 670 mg, raw product obtained as described above) and sodium acetate (164 mg, 2.0 mmol). Yellow needles, mp 165–170°C, 131 mg (24%); IR (KBr): 3192 cm^-1^ (NH), 1671 cm^-1^ (C = O); ^1^H NMR (DMSO-d_6_, 400 MHz): δ 1.26 (t, 3H, *J* = 7.4 Hz, ethyl-CH_3_), 2.84 (q, 2H, *J* = 7.5, ethyl-CH_2_), 4.44 (s, 2H, CH_2_), 7.16 (d, 2H, *J* = 8.1 Hz), 7.26–7.36 (m, 1H), 7.41–7.53 (m, 4H), 7.67 (d, 1H, *J* = 8.1 Hz), 8.40 (s, 1H, pyrrolidone-NH), 11.56 (s, 1H, indole-NH); ^13^C NMR (DMSO-d_6_, 101 MHz): δ 15.15 (CH_3_); 19.04, 45.52 (CH_2_); 114.12, 121.60, 125.77, 128.62 (2C), 129.06 (2C) (CH); 112.45, 115.78, 127.33, 129.52, 135.11, 138.75, 139.28, 170.57 (C); C_18_H_16_N_2_O (276.3); MS (EI) *m/z* 276.1 [M]^+.^, HRMS (EI) *m/z* [M]^+.^ calcd 276.12571, obsd 276.12606.

*5-Bromo-3-phenyl-6*,*7-dihydropyrrolo[3*,*4-g]indol-8(1H)-one (****12a****)*. From phenylacetaldehyde dimethylacetal (500 μL, 3.00 mmol), 2-(7-bromo-3-oxoisoindolin-4-yl)hydrazin-1-ium chloride (**11b**, 1035 mg, raw product obtained as described above) and sodium acetate (246 mg, 3.0 mmol). Yellow solid, mp 226–227°C, 270 mg (28%); IR (KBr): 3449 cm^-1^, 3424 cm^-1^ (NH), 1708 cm^-1^, 1674 cm^-1^ (C = O); ^1^H NMR (DMSO-d_6_, 400 MHz): 4.39 (s, 2H, CH_2_), 7.24–7.34 (m, 1H), 7.41–7.51 (m, 2H), 7.65–7.73 (m, 3H), 8.18 (s, 1H), 8.74 (s, 1H, pyrrolidone-NH), 12.04 (d, 1H, *J* = 2.9 Hz); ^13^C NMR (DMSO-d_6_, 101 MHz): δ 46.40 (CH_2_); 124.60, 125.47, 125.95, 126.93 (2C), 128.93 (2C) (CH); 107.41, 116.02, 118.30, 127.62, 130.07, 134.49, 138.27, 169.51 (C); C_16_H_11_BrN_2_O (327.18); MS (EI): *m/z* 326.0 [M]^+.^.

*5-Bromo-2-methyl-3-phenyl-6*,*7-dihydropyrrolo[3*,*4-g]indol-8(1H)-one (****12b****)*. From phenylacetone (230 μL, 1.70 mmol), 2-(7-bromo-3-oxoisoindolin-4-yl)hydrazin-1-ium chloride (**11b**, 587 mg, raw product obtained as described above) and sodium acetate (139 mg, 1.7 mmol). Light yellow needles, mp 255–256°C, 113 mg (19%); IR (KBr): 3418 cm^-1^, 3216 cm^-1^ (NH), 1691 cm^-1^ (C = O); ^1^H NMR (DMSO-d_6_, 400 MHz): δ 2,50 (s, 3H, CH_3_), 4.35 (s, 2H, CH_2_), 7.27–7.38 (m, 1H), 7.45–7.54 (m, 4H), 7.79 (s, 1H), 8.68 (s, 1H, pyrrolidone-NH), 11.85 (s, 1H, indole-NH); ^13^C NMR (DMSO-d_6_, 101 MHz): δ 12.15 (CH_3_), 46.30 (CH_2_), 123.12, 125.93, 128.72 (2C), 128.92 (2C) (CH), 106.81, 112.56, 117.42, 128.66, 129.69, 134.35, 135.26, 137.10, 169.63 (C); C_17_H_13_BrN_2_O (341.2); MS (EI) *m/z* 340.0 [M]^+.^, HRMS (EI) *m/z* [M]^+.^ calcd 340.02058, obsd 340.02053.

*5-Chloro-3-phenyl-6*,*7-dihydropyrrolo[3*,*4-g]indol-8(1H)-one (****12c****)*. From phenylacetaldehyde dimethylacetal (415 μL, 2.50 mmol), 2-(7-chloro-3-oxoisoindolin-4-yl)hydrazin-1-ium chloride (**11c**, 640 mg, raw product obtained as described above) and sodium acetate (205 mg, 2.5 mmol). White solid, mp 208–210°C, 101 mg (15%); ^1^H NMR (DMSO-d_6_, 400 MHz): 4.45 (s, 2H, CH_2_), 7.28 (tt, 1H, *J* = 1.0 Hz, 7.4 Hz), 7.46 (tt, 2H, *J* = 1.9 Hz, 7.6 Hz), 7.65–7.73 (m, 3H), 8.04 (s, 1H), 8.71 (s, 1H, pyrrolidone-NH), 12.01 (br s, 1H, indole-NH); ^13^C NMR (DMSO-d_6_, 101 MHz): δ 44.79 (CH_2_); 121.67, 125.57, 125.92, 126.89 (2C), 128.90 (2 C) (CH), 116.15, 118.05, 119.26, 127.05, 129.67, 134.50, 136.44, 169.42 (C); C_16_H_11_ClN_2_O (282.72); MS (EI): *m/z* 282.1 [M]^+^.

*7-Methyl-3-phenyl-6*,*7-dihydropyrrolo[3*,*4-g]indol-8(1H)-one (****12d****)*. From phenylacetaldehyde dimethylacetal (166 μL, 1.00 mmol), 2-(2-methyl-3-oxoisoindolin-4-yl)hydrazin-1-ium chloride (**11d**, 455 mg, raw product obtained as described above) and sodium acetate (82 mg, 1.0 mmol). Off-white powder, mp. 265–267°C, 82 mg (31%); IR (KBr): 3306 cm^-1^(NH), 1659 cm^-1^ (C = O), 1600 cm^-1^; ^1^H NMR (DMSO-d_6_, 400 MHz): δ 3.13 (s, 3H, CH_3_), 4.42 (s, 2H, CH_2_), 7.24–7.30 (m, 2H), 7.41–7.48 (m, 2H), 7.62 (d, 1H, *J* = 2.6 Hz), 7.67–7.74 (m, 2H), 8.06 (d, 1H, *J* = 8.2 Hz), 11.78 (s, 1H, indole-NH); ^13^C NMR (DMSO-d_6_, 101 MHz): δ 28.86 (CH_3_); 52.06 (CH_2_); 114.32, 122.45, 124.07, 125.63, 126.84, 128.81 (CH); 116.39, 130.54, 135.19, 136.91, 167.60 (C); C_17_H_14_N_2_O (262,3); MS (EI): *m/z* 262.1 [M]^+^.

#### Synthesis of compounds 8b, 8f, 8j

PdCl_2_(MeCN)_2_ (0.13 equiv), *p*-benzoquinone (0.6 equiv) were suspended in heated *tert*-butanol (5 mL) at 85°C. Subsequently water (0.5 equiv) and the respective styrene (0.50 equiv) were added and then the mixture was stirred at 85°C for 1 h. Afterwards the mixture was cooled on ice until solid to stop the reaction. In the next step the mixture was heated again to 30°C and subsequently the 2-(3-oxoisoindolin-4-yl)hydrazin-1-ium chloride **11a** suspension (raw product obtained as described above, H_2_SO_4_ (98%, 0.88 mL), water (1.46 mL) and ethanol (4.39 mL)) was added. The mixture was stirred at 50°C for 2.5 h. After completion of the reaction, water (20 mL) was added and then the suspension was extracted with ethyl acetate (5 x 15 mL). The combined organic phases were dried over Na_2_SO_4_ and the solvent was evaporated under vacuum mounting the mixture on silica gel (1.5 g). Purification was done by column chromatography (toluene/ethyl acetate/acetic acid(5/5/0.5) and recrystallization from ethanol.

*3-(2-Chlorophenyl)-6*,*7-dihydropyrrolo[3*,*4-g]indol-8(1H)-one (****8b****)*. From PdCl_2_(MeCN)_2_ (7 mg, 25 μmol), *p*-benzoquinone (125 mg, 1.18 mmol), 2-chlorostyrene (127 μL, 1.00 mmol) and 2-(3-oxoisoindolin-4-yl)hydrazin-1-ium chloride (**11a**, 670 mg, raw product obtained as described above). Beige powder, mp 234–235°C, 58 mg (21%); IR (KBr): 3439cm^-1^(NH), 1668cm^-1^ (C = O); ^1^H NMR (DMSO-d_6_, 600 MHz): 4.42 (s, 2H, CH_2_), 7.23 (d, 1H, *J* = 8.1 Hz), 7.33–7.39 (m, 1H), 7.43 (dt, 1H, *J* = 1.4 Hz, 7.5 Hz), 7.51 (d, 1H, *J* = 2.6 Hz), 7.57 (dd, 1H, *J* = 1.7 Hz, 7.6 Hz), 7.60 (dd, 1H, *J* = 1.3 Hz, 8.0 Hz), 7.66 (d, 1H, *J* = 8.1 Hz), 8.46 (s, 1H, pyrrolidone-NH), 8.87 (br s, 1H, indole-NH); ^13^C NMR (DMSO-d_6_, 151 MHz): δ 45.55 (CH_2_); 114.49, 122.91, 125.64, 127.20, 129.88, 131.93 (CH); 113.27, 116.51, 126.14, 127.99, 129.84, 132.07, 133.35, 139.54, 170.27 (C); C_16_H_11_ClN_2_O (282.7); MS (EI): *m/z* 282.1 [M]^+.^.

*3-(3-Methoxyphenyl)-6*,*7-dihydropyrrolo[3*,*4-g]indol-8(1H)-one (****8f****)*. From PdCl_2_(MeCN)_2_ (13 mg, 50 μmol), *p*-benzoquinone (249 mg, 2.36 mmol), 3-methoxystyrene (275 μL, 2.00 mmol) and 2-(3-oxoisoindolin-4-yl)hydrazin-1-ium chloride (**11a**, 1340 mg, raw product obtained as described above). Red solid, mp 207–208°C, 64 mg (23%); IR (KBr): 3397 cm^-1^ and 3246 cm^-1^ (NH), 1670 cm^-1^ (C = O); ^1^H NMR (DMSO-d_6_, 400 MHz): δ 3.83 (s, 3H, CH_3_), 4.47 (s, 2H, CH_2_), 6.84 (dd, 1H, *J* = 8.2, 2.6), 7.20–7.38 (m, 4H), 7.64 (d, 1H, *J* = 2.6 Hz), 8.07(d, 1H, *J* = 8.2 Hz), 8.43 (s, 1H, pyrrolinone-NH), 11.77 (s, 1H, indole-NH); ^13^C NMR (DMSO-d_6_, 101 MHz): δ 54.72 (CH_3_); 45.35 (CH_2_); 111.16, 111.85, 114.48, 118.95, 122.54, 123.93, 129.53 (CH); 116.26, 116.57, 125.26, 130.83, 136.63, 139.54, 159.66 (C); C_17_H_14_N_2_O_2_ (278.3); MS (EI): *m/z* 278.1 [M]^+.^.

*3-(3-Nitrophenyl)-6*,*7-dihydropyrrolo[3*,*4-g]indol-8(1H)-one (****8j****)*. From PdCl_2_(MeCN)_2_ (3 mg, 13 μmol), *p*-benzoquinone (62 mg, 0.6 mmol), 3-nitrostyrene (70 μL, 0.50 mmol) and 2-(3-oxoisoindolin-4-yl)hydrazin-1-ium chloride (**11a**, 335 mg, raw product obtained as described above). Yellow solid, mp 237–238°C (decomposition), 76 mg (26%); IR (KBr): 3401 cm^-1^, 3280 cm^-1^ (NH), 1686 cm^-1^ (C = O); ^1^H NMR (DMSO-d_6_, 400 MHz): δ 4.49 (s, 2H, CH_2_), 7.35 (d, 1H, *J* = 8.2 Hz), 7.73 (t, 1H, *J* = 8.0 Hz), 7.90 (d, 1H, *J* = 2.7 Hz), 8.10 (m, 2H), 8.20 (dt, 1H, *J* = 1.3 Hz, 7.9 Hz), 8.48 (t, 1H, *J* = 2.0 Hz), 8.51 (s, 1H, pyrrolinone-NH), 12.04 (s, 1H, indole-NH); ^13^C NMR (DMSO-d_6_, 101 MHz): δ 45.7 (CH_2_); 115.4, 120.1, 120.6, 122.3, 125.7, 130.3, 133.1 (CH); 114.1, 116.9, 124.9, 131.0, 137.1, 140.0, 148.4, 170.3 (C); C_16_H_11_N_3_O_3_(293.8); MS: (EI) *m/z* 293.1 [M]^+.^; HRMS (EI) *m/z* [M]^+.^ calcd 293.07949, obsd 293.07913. HPLC: 98.7% at 256 nm and 98.9% at 280 nm; t_N_ = 3.6 min, t_M_(DMSO) = 1.06 min (ACN/H_2_O = 60/40), HPLC (Gradient elution: (ACN/H_2_O) 0-10 min (10/90→70/30, linear), 10 min-10.5 min (70/30→90/10, linear), 10.5 min-16.5 min (90/10)): 89.4% at 254 nm and 97.2% at 280 nm; t_N_ = 10.6 min. λ_max_ = 232 nm, 269 nm.

*3-(3-Hydroxyphenyl)-6*,*7-dihydropyrrolo[3*,*4-g]indol-8(1H)-one (****8g****)*. 3-(3-Methoxyphenyl)-6,7-dihydropyrrolo[3,4-*g*]indol-8(1*H*)-one **8f** (167 mg, 0.60 mmol) was dissolved in dry CH_2_Cl_2_ (15 mL) under nitrogen atmosphere. Boron tribromide solution in dichloromethane (3 mmol, 3 mL, c = 1 M) was added and the mixture was stirred at RT under N_2_-atmosphere for 1 h. After adding water (28 mL) and stirring for another hour, the precipitate was filtered under vacuum. The filtrate was partitioned between water (28 mL) and ethyl acetate (5 x 30 mL). The precipitate was dissolved in the dried organic phases, the solvent evaporated under vacuum and the precipitate recrystallized from ethanol 80%.

Brown solid, mp 235–236°C (decomposition), 53 mg (33%); IR (KBr): 3386 cm^-1^ (NH), 3222 cm^-1^ (NH), 1656 cm^-1^ (C = O); ^1^H NMR (DMSO-d_6_, 400 MHz): δ 4.47 (s, 2H, CH_2_), 6.67 (ddd, 1H, *J* = 1.1 Hz, 2.3 Hz, 8.1 Hz), 7.11 (m, 2H), 7.23 (t, 1H, *J* = 8.0 Hz), 7.27 (d, 1H, *J* = 8.2 Hz), 7.53 (d, 1H, *J* = 2.6 Hz), 8.04 (d, 1H, *J* = 8.2 Hz), 8.45 (s, 1H, pyrrolidone-NH), 9.39 (s, 1H, phenol-OH), 11.73 (s, 1H, indole-NH); ^13^C NMR (DMSO-d_6_, 101 MHz): δ 45.6 (CH_2_), 112.7, 113.7, 114.6, 117.7, 122.8, 123.7, 129.8 (CH), 116.5, 116.6, 125.3, 130.8, 136.4, 139.5, 157.7, 170.5 (C); C_16_H_12_N_2_O_2_ (264.3); MS (EI) *m/z* 264.1 [M]^+.^.

*3-(8-Oxo-1*,*6*,*7*,*8-tetrahydropyrrolo[3*,*4-g]indol-3-yl)phenyl acetate (****8h****)*. To a solution of 3-(3-hydroxyphenyl)-6,7-dihydropyrrolo[3,4-*g*]indol-8(1*H*)-one **8g** (151 mg, 0.57 mmol) in pyridine (7 mL) acetic anhydride (60 μL, 0.63 mmol) and 4-dimethylaminopyridine (4-DMAP, cat, ca. 4 mg) were added. After stirring the mixture for 2 h at room temperature the solvent was evaporated under vacuum. The crude product was dissolved in ethyl acetate (20 mL) and extracted with water (20 mL). The aqueous phase was further extracted with ethyl acetate (3 x 20 mL). The combined organic phases were washed with water (2 x 50 mL), dried over Na_2_SO_4_ and the solvent was evaporated under vacuum. Purification by column chromatography (ethyl acetate/ethanol/trimethylamine (6/2/2)) and recrystallization from ethanol.

Yellow solid, mp 185–186°C, 89 mg (29%); IR (KBr): 3337 cm^-1^ (NH), 3194 cm^-1^ (NH), 1741 cm^-1^ (C = O), 1678 cm^-1^ (C = O); ^1^H NMR (DMSO-d_6_, 600 MHz): δ 2.30 (s, 3H, CH_3_), 4.48 (s, 2H, CH_2_), 7.01 (ddd, 1H, *J* = 1.0 Hz, 2.3 Hz, 8.0 Hz), 7.29 (d, 1H, *J* = 8.1 Hz), 7.45–7.49 (m, 2H), 7.59–7.62 (m, 1H), 7.67 (d, 1H, *J* = 2.3 Hz), 8.07 (d, 1H, *J* = 8.2), 8.47 (s, 1H, pyrrolidone-NH), 11.85 (s, 1H, indole-NH); ^13^C NMR (DMSO-d_6_, 151 MHz): δ 21.13 (CH_3_), 45.55 (CH_2_), 114.83, 118.77, 119.82, 122.58, 123.99, 124.44, 129.73 (CH), 115.27, 116.59, 124.97, 130.79, 136.64, 139.60, 150.91, 169.21, 170.28 (C); C_18_H_14_N_2_O_3_ (306.3); MS (EI) *m/z* 306.1 [M]^+.^, HRMS (EI) *m/z* [M]^+.^ calcd 306.09989, obsd 306.10020. HPLC: 94.3% at 254 nm and 96,4% bei 280 nm; t_N_ = 4.6, t_M_(DMSO) = 1,06 min (ACN/H_2_O = 40/60); HPLC (Gradient elution: (ACN/H_2_O) 0-10 min (10/90→70/30, linear), 10 min-10.5 min (70/30→90/10, linear), 10.5 min-16.5 min (90/10)): 93.4% at 254 nm; t_N_ = 9.44 min. λ_max_ = 270 nm, 307 nm.)

#### Synthesis of compounds 15a-15e

In a round-bottom flask, 3-phenyl-6,7-dihydropyrrolo[3,4-*g*]indol-8(1*H*)-one **8a** (1 equiv) was dissolved in acetone (10 mL). At room temperature potassium *tert*-butoxide (1 equiv) was added and the mixture was stirred for one hour. The respective halogen alkane (10 equiv) was added and stirring continued for another 24 h at room temperature. Purification of the crude product was accomplished by column chromatography on silica gel (toluene/ethyl acetate/acetic acid (8/1/0.5)) and recrystallization from toluene/hexane (2+1).

*1-Methyl-3-phenyl-6*,*7-dihydropyrrolo[3*,*4-g]indol-8(1H)-one (****15a****)*. From 3-phenyl-6,7-dihydropyrrolo[3,4-*g*]indol-8(1*H*)-one **8a** (161 mg, 0.65 mmol), potassium *tert*-butoxide (73 mg, 0.7 mmol) and iodomethane (405 μL, 6.50 mmol). Yellow solid, mp 255–256°C, 75 mg (44%); IR (KBr): 3208 cm^-1^ (NH), 1686 cm^-1^ (C = O), 1639 cm^-1^, 1600 cm^-1^(C = C, aromatic); ^1^H NMR (DMSO-d_6_, 400 MHz): δ 4.40 (s, 3H, CH_3_), 4.46 (s, 2H, CH_2_), 7.28 (m, 2H), 7.45 (m, 2H), 7.64 (m, 3H), 8.04 (m, 1H), 8.51 (s, 1H, pyrrolinone-NH); ^13^C NMR (DMSO-d_6_, 101 MHz): δ 37.69 (CH_3_); 45.32 (CH_2_); 114.84, 123.04, 125.80, 127.04, 128.82, 128.96 (CH); 116.63, 117.12, 126.30, 132.76, 134.75, 141.32, 170.11 (C); C_17_H_14_N_2_O (262.3); MS (EI) *m/z* 262.1 [M]^+.^.

*1-Ethyl-3-phenyl-6*,*7-dihydropyrrolo[3*,*4-g]indol-8(1H)-one (****15b****)*. From 3-phenyl-6,7-dihydropyrrolo[3,4-*g*]indol-8(1*H*)-one **8a** (248 mg, 1.00 mmol), potassium *tert*-butoxide (112 mg, 1.00 mmol) and iodoethane (805 μL, 10.0 mmol). Yellow needles, mp 198–200°C, 132 mg (48%); IR (KBr): 3189 cm^-1^ (NH), 1679 cm^-1^ (C = O); ^1^H NMR (DMSO-d_6_, 400 MHz): δ 1.37 (t, 3H, *J* = 7.0 Hz, ethyl-CH_3_), 4.46 (s, 2H, CH_2_), 5.00 (q, 2H, *J* = 7.0 Hz, ethyl-CH_2_), 7.22–7.33 (m, 2H), 7.41–7.51 (m, 2H), 7.61–7.69 (m, 2H), 7.72 (s, 1H), 8.05 (d, 1H, *J* = 8.2 Hz), 8.51 (s, 1H, pyrrolidone-NH); ^13^C NMR (DMSO-d_6_, 101 MHz): δ 17.24 (CH_3_); 44.60, 45.30 (CH_2_); 114.82, 123.14, 125.78, 127.07, 127.49, 128.81 (CH); 116.89, 116.92, 126.29, 131.76, 134.84, 141.34, 170.11 (C); C_18_H_16_N_2_O (276.3); MS (EI) *m/z* (%) 276.1 [M]^+.^.

*2-(8-Oxo-3-phenyl-7*,*8-dihydropyrrolo[3*,*4-g]indol-1(6H)-yl)acetamide (****15c****)*. From 3-phenyl-6,7-dihydropyrrolo[3,4-*g*]indol-8(1*H*)-one **8a** (186 mg, 0.75 mmol), potassium *tert*-butoxide (96 mg, 0.75 mmol) and 2-bromoacetamide (1035 mg, 7.50 mmol). Divergent from the general intructions purification was conducted by filtration through silica gel (3 cm) and product elution with ethanol (96%, 300 mL). Light yellow solid, mp 284–285°C (decomposition), 35 mg (15%);^1^H NMR (DMSO-d_6_, 600 MHz): δ 4.46 (s, 2H, pyrrolidinon-CH_2_), 5.73 (s, 2H, CH_2_), 7.04 (s, 1H, amide-NH), 7.25–7.32 (m, 2H), 7.42 (s, 1H, amide-NH), 7.44–7.50 (m, 2H), 7.57 (s, 1H), 7.63–7.68 (m, 2H), 8.06 (d, 1H, *J* = 8.1 Hz), 8.52 (s, 1H, pyrrolidone-NH); ^13^C NMR (DMSO-d_6_, 101 MHz): δ 45.39, 52.22 (CH_2_); 114.79, 122.99, 125.76, 126.94 (2C), 128.78 (2C), 128.97 (CH); 116.82, 116.99, 126.14, 133.37, 134.66, 141.14, 170.24, 170.30 (C); C_18_H_15_N_3_O_2_ (305.3); MS (EI) *m/z* 305.1 [M]^+.^, HRMS (EI) *m/z* [M]^+.^ calcd 305.11588, obsd 305.11515.

*Ethyl 2-(8-oxo-3-phenyl-7*,*8-dihydropyrrolo[3*,*4-g]indol-1(6H)-yl)acetate (****15d****)*. From 3-phenyl-6,7-dihydropyrrolo[3,4-*g*]indol-8(1*H*)-one **8a** (198 mg, 0.80 mmol), potassium *tert*-butoxide (90 mg, 0.8 mmol) and ethyl 2-bromoacetate (885 μL, 8.00 mmol). Yellow solid, mp 226-227°C, 69 mg (26%); IR (KBr): 3234 cm^-1^ (NH), 1739 cm^-1^ (C = O), 1672 cm^-1^(C = O), 1634 cm^-1^ (C = O); ^1^H NMR (DMSO-d_6_, 400 MHz): 1.21 (t, 3H, *J* = 7.1 Hz, CH_3_), 4.13 (q, 2H, *J* = 7.1 Hz, Ethyl-CH_2_), 4.46 (s, 2H, pyrrolidone-CH_2_), 5.80 (s, 2H, CH_2_), 7.24–7.36 (m, 2H), 7.42–7.52 (m, 2H), 7.60–7.70 (m, 3H), 8.07 (d, 1H, *J* = 8.2 Hz), 8.52 (s, 1H, pyrrolidone-NH); ^13^C NMR (DMSO-d_6_, 101 MHz): δ 14.06 (CH_3_); 45.51, 51.52, 60.48 (CH_2_); 115.20, 123.13, 126.00, 127.04, 128.65, 128.89 (CH); 117.07, 117.20, 126.35, 133.01, 134.51, 141.23, 169.58, 170.17 (C); C_20_H_18_N_2_O_3_ (334.4); MS (EI) *m/z* (%) 334.1 [M]^+.^, HRMS (EI) *m/z* [M]^+.^ calcd 334.13119, obsd 334.13053.

*1*,*7-Dimethyl-3-phenyl-6*,*7-dihydropyrrolo[3*,*4-g]indol-8(1H)-one (****15e****)*. From 3-phenyl-6,7-dihydropyrrolo[3,4-*g*]indol-8(1*H*)-one **8a** (124 mg, 0.50 mmol), potassium *tert*-butoxide (73 mg, 0.7 mmol) and iodomethane (315 μL, 5.00 mmol) dissolved in dry THF (10 mL) instead of acetone. Purification by preparative HPLC (ACN/H_2_O (60/40)). Yellow solid, mp 151–152°C, 36 mg (27%); IR (KBr): 3426 cm^-1^ (NH), 1661 cm^-1^ (C = O); ^1^H NMR (DMSO-d_6_, 400 MHz): δ 3.12 (s, 3H, CH_3_), 4.40 (s, 3H, CH_3_), 4.54 (s, 2H, CH_2_), 7.28 (m, 2H), 7.46 (m, 2H), 7.65 (m, 3H), 8.04 (d, 1H, *J* = 8.3); ^13^C NMR (DMSO-d_6_, 101 MHz): δ 29,14, 37,59 (CH_3_); 51,71 (CH_2_); 114,39, 122,67, 125,77, 126,99, 128,79, 129,08 (CH); 116,57, 116,92, 126,46, 132,36, 134,71, 138,48, 167,21 (C); C_18_H_16_N_2_O (276.3); MS (EI) *m/z* 276.1 [M]^+.^, HRMS (EI) m/z [M]^+.^ calcd 276.12571, obsd 276.12532.

*2-Bromo-3-phenyl-6*,*7-dihydropyrrolo[3*,*4-g]indol-8(1H)-one (****16****)*. 3-Phenyl-6,7-dihydropyrrolo[3,4-*g*]indol-8(1*H*)-one (**8a**, 124 mg, 0.50 mmol) was dispersed in CH_2_Cl_2_/acetic acid (6 mL + 2 mL) under nitrogen atmosphere at < 10°C. NBS (98 mg, 0.6 mmol) was added and the mixture was stirred for 1.5 h at room temperature. The solvent was evaporated under vacuum, the crude product was dissolved in ethyl acetate (30 mL) and the solution was extracted with potassium hydroxide solution (10%, 3 x 15 mL). After washing the organic phase with water (30 mL) it was dried over Na_2_SO_4_ and the solvent was evaporated under vacuum. Purification by recrystallization from ethanol 96%.

Light yellow solid, mp 248–250°C, 47 mg (29%); IR (KBr): 3409 cm^-1^(NH), 1691 cm^-1^ (C = O); ^1^H NMR (DMSO-d_6_, 400 MHz): δ 4.46 (s, 2H, CH_2_), 7.24 (d, 1H, *J* = 8.2 Hz), 7.33–7.42 (m, 1H), 7.48–7.55 (m, 2H), 7.55–7.21 (m, 2H), 7.73 (d, 1H, *J* = 8.0 Hz), 8.49 (br s, 1H, pyrrolidone-NH), 12.52 (br s, 1H, indole-NH); ^13^C NMR (DMSO-d_6_, 101 MHz): δ 45.53 (CH_2_), 115.33, 121.66, 126.71, 128.59, 129.20 (CH), 109.14, 115.79, 116.14, 126.62, 130.25, 133.08, 140.09, 169.84 (C); C_16_H_11_BrN_2_O (327.2); MS (EI) *m/z* 326.0[M]^+.^.

#### Synthesis of compounds 17a-17c

5-Bromo-3-phenyl-6,7-dihydropyrrolo[3,4-*g*]indol-8(1*H*)-one (**12a**) (1 equiv), tetrakistriphenylphosphinpalladium(0) (0.4 equiv), Cs_2_CO_3_ (3 equiv) and the respective phenylboronic acid (1.5 equiv) were suspended in toluene/ethanol (2 mL + 1 mL) in a microwave reactor. The reaction was executed using the following parameters: ramp time: 5 min, reaction time: 20 min at 150°C, irradiation: 200 W. After filtering the crude product over silica gel (3 cm) the solvent was evaporated under vacuum. Purification was done by recrystallization from ethanol or by preparative HPLC.

*3*,*5-Diphenyl-6*,*7-dihydropyrrolo[3*,*4-g]indol-8(1H)-one (****17a****)*. From 5-bromo-3-phenyl-6,7-dihydropyrrolo[3,4-*g*]indol-8(1*H*)-one (**12a**) (131 mg, 0.4 mmol), Tetrakistriphenylphosphin-palladium(0) (4 mg, 3.4 μmol) and Cs_2_CO_3_ (254 mg, 0.78 mmol) and phenylboronic acid (98 mg, 0.8 mmol). White yellow solid, mp 262–263°C, 18 mg (14%); IR (KBr): 3417 cm^-1^ (NH), 1688 cm^-1^ (C = O); ^1^H NMR (DMSO-d_6_, 600 MHz): δ 4.56 (s, 2H, CH_2_), 7.27 (tt, 1H, *J* = 1.2 Hz, 7.3 Hz), 7.39 (tt, 1H, *J* = 1.2 Hz, 7.4 Hz), 7.43–7.52 (m, 4H), 7.63–7.66 (m, 2H), 7.67 (d, 1H, *J* = 2.5 Hz), 7.71–7.77 (m, 2H), 8.02 (s, 1H), 8.58 (s, 1H, pyrrolidone-NH), 11.85 (d, 1H, *J* = 2.0 Hz, indole-NH); ^13^C NMR (DMSO-d_6_, 151 MHz): δ 45.62 (CH_2_); 122.29, 124.63, 125.61, 126.85 (2C), 128.21 (2C), 128.65 (2C), 128.81 (2C)(CH); 116.53, 116.87, 126.07, 128.71, 130.39, 135.04, 137.16, 139.74, 170.18 (C); C_22_H_16_N_2_O (324.4); MS (EI) *m/z* 324.1 [M]^+.^, HRMS (EI) *m/z* [M]^+.^ calcd 324.12571, obsd 324.12541.

*5-(2-Fluorophenyl)-3-phenyl-6*,*7-dihydropyrrolo[3*,*4-g]indol-8(1H)-one (****17b****)*. From 5-bromo-3-phenyl-6,7-dihydropyrrolo[3,4-*g*]indol-8(1*H*)-one (**12a**) (196 mg, 0.60 mmol), tetrakis-triphenylphosphinpalladium(0) (28 mg, 0.024 mmol), Cs_2_CO_3_ (586 mg, 1.80 mmol) and 2-fluorophenylboronic acid (126 mg, 0.90 mmol). Yellow solid, mp 136-139°C, 11mg (5.4%); IR (KBr): 3417 cm^-1^ (NH), 3262 cm^-1^ (NH), 2922 cm^-1^, 1685 cm^-1^ (C = O); ^1^H NMR (DMSO-d_6_, 600 MHz): δ 4.36 (s, 2H, CH_2_), 7.24–7.28 (m, 1H), 7.30–7.39 (m, 2H), 7.41–7.51 (m, 3H), 7.61 (td, 1H, *J* = 1.8 Hz, 7.72 Hz), 7.70 (d, 1H, *J* = 2.6 Hz), 7.70–7.74 (m, 2H), 7.99 (s, 1H), 8.55 (br s, 1H, pyrrolidone-NH), 11.93 (d, 1H, *J* = 2,6 Hz, indole-NH); ^13^C NMR (DMSO-d_6_, 151 MHz): δ (ppm) = 45.21 (d, ^5^J_C,F_ = 4.47 Hz, C-C-C-C-C-F) (CH_2_); 115.80 (d, ^2^J_C,F_ = 22.44, Hz, C-C-F), 123.66, 124.71 (d, ^4^J_C,F_ = 3.28 Hz, C-C-C-C-F), 124.78, 125.70, 126.86 (2C), 128.83 (2C), 129.55 (d, ^3^J_C,F_ = 8.13 Hz, C-C-C-F), 131.66 (d, ^4^J_C,F_ = 3.29 Hz, C-C-C-C-F) (CH); 116.52, 116.69, 122.36, 125.76, 126.76 (d, ^2^J_C,F_ = 15.46 Hz, C-C-F), 130.61, 134.91, 138.11, 159.05 (d, ^1^J_C,F_ = 244.03 Hz, C-F), 170.09 (C); C_22_H_15_FN_2_O (342.4); MS (EI) *m/z* 342.1 [M]^+.^, HRMS (EI) *m/z* [M]^+.^ calcd 342.11629, obsd 342.11663.

*5-(3-Fluorophenyl)-3-phenyl-6*,*7-dihydropyrrolo[3*,*4-g]indol-8(1H)-one (****17c****)*. From 5-bromo-3-phenyl-6,7-dihydropyrrolo[3,4-*g*]indol-8(1*H*)-one (**12a**) (98 mg, 0.3 mmol), tetrakistriphenylphosphinpalladium(0) (14 mg, 0.012 mmol), Cs_2_CO_3_ (293 mg, 0.90 mmol) and 3-fluorophenylboronic acid (63 mg, 0.3 mmol). Grey solid, mp 246–247°C, 32 mg (31%); IR (KBr): 3416 cm^-1^ (NH), 1690 cm^-1^ (C = O); ^1^H NMR (DMSO-d_6_, 600 MHz): δ 4.48 (s, 1H, CH_2_), 7.19–7.24 (m, 1H), 7,27 (tt, 1H, *J* = 1.2 Hz, 7.4 Hz), 7.42–7.47 (m, 2H), 7.48–7.55 (m, 3H), 7.68 (d, 1H, *J* = 2.6), 7.73–7.78 (m, 2H), 8.05 (s, 1H), 8.61 (br s, 1H, pyrrolidone-NH), 11.89 (d, 1H, *J* = 2.7 Hz, indole-NH); ^13^C NMR (DMSO-d_6_, 151 MHz): δ 45.53 (CH_2_); 113.68 (d, ^2^J_C,F_ = 20.75 Hz, C-C-F), 114.98 (d, ^2^J_C,F_ = 21.53 Hz, C-C-F), 122,53, 124.48 (d, ^4^J_C,F_ = 2.55 Hz, C-C-C-C-F), 124.82, 125.68, 126.92 (2C), 128.84 (2C), 130.55 (d, ^3^J_C,F_ = 8.66 Hz, C-C-C-F) (CH); 116.69, 116.95, 126.04, 127.41 (d, ^4^J_C,F_ = 1.93 Hz, C-C-C-C-F), 130.63, 134.96, 137.16, 142.22 (d, ^3^J_C,F_ = 7.95 Hz, C-C-C-F), 162.35 (d, ^1^J_C,F_ = 243.55 Hz, C-F), 170.10 (C); C_22_H_15_FN_2_O (342.4); MS (EI): *m/z* 342.1 [M]^+.^, HRMS (EI) *m/z* [M]^+.^ calcd 342.11629, obsd 342.11679.

## Supporting information

S1 Fig Result of a docking experiment with 8c in CLK1. (PDF)

S1 Table Results from a preliminary screening for CLK1 inhibitors. (PDF)

S2 Table Results of crystal violet assays with KuWal151. (PDF)
