# Supplementary material for: Molecular structures of cdc2-like kinases in complex with a new inhibitor chemotype
Source: PLoS One. 2018 May 3;13(5):e0196761. doi: 10.1371/journal.pone.0196761 (PMC5933782; doi:10.1371/journal.pone.0196761)
Supplement: S1 Fig — (PDF) [file pone.0196761.s001.pdf]

Molecular structures of cdc2-like kinases in complex with a new inhibitor chemotype

Anne Walter, Apirat Chaikuad, Renate Helmer, Nadège Loaëc, Lutz Preu, Ingo Ott, Stefan Knapp, Laurent Meijer, Conrad Kunick

Supporting information

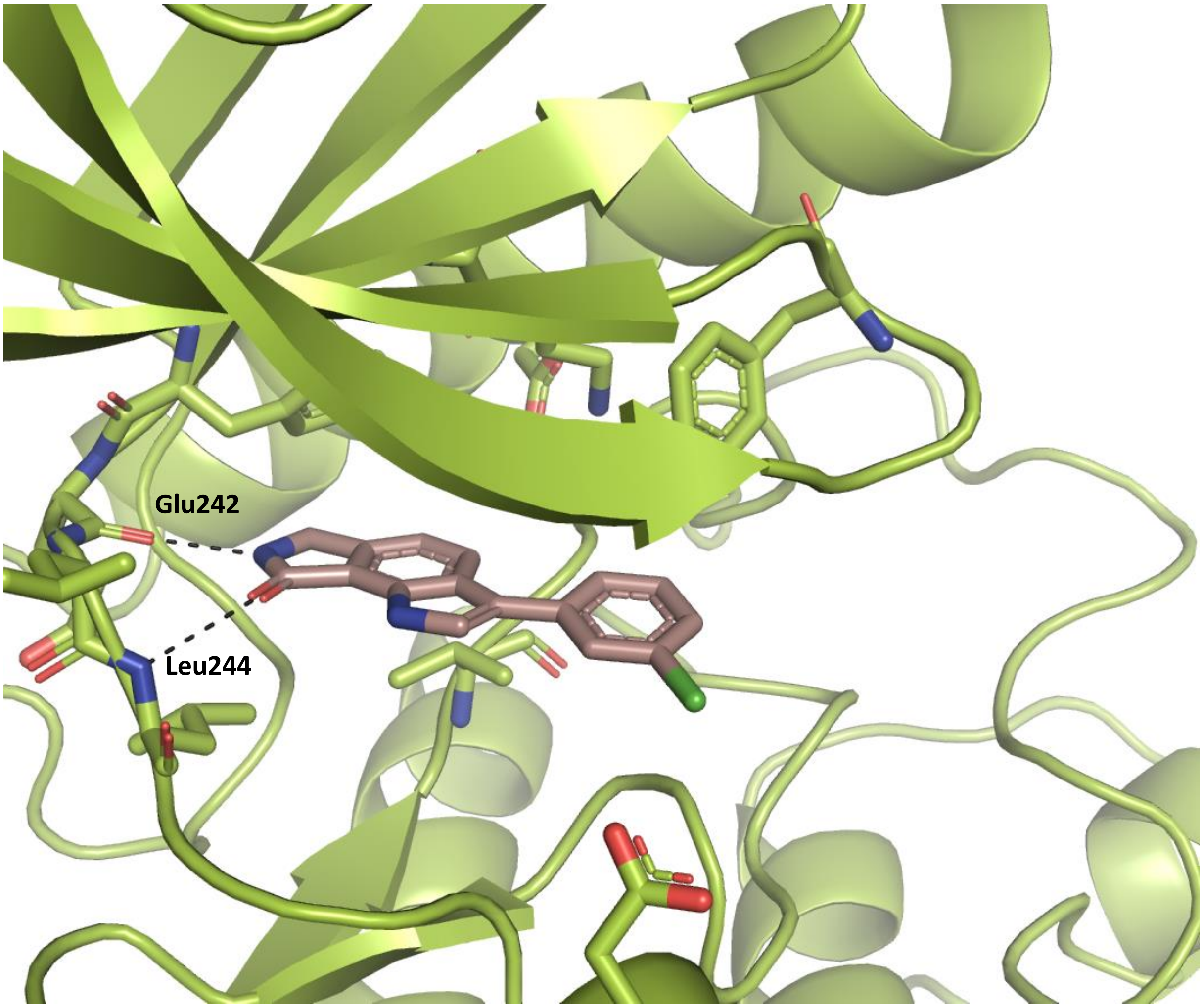

S1 Fig. Result of a docking experiment with **8c** in CLK1 (PDB: 1Z57).
