# Supplementary material for: Molecular structures of cdc2-like kinases in complex with a new inhibitor chemotype
Source: PLoS One. 2018 May 3;13(5):e0196761. doi: 10.1371/journal.pone.0196761 (PMC5933782; doi:10.1371/journal.pone.0196761)
Supplement: S1 Table — (PDF) [file pone.0196761.s002.pdf]

Molecular structures of cdc2-like kinases in complex with a new inhibitor chemotype

Anne Walter, Apirat Chaikuad, Renate Helmer, Nadège Loaëc, Lutz Preu, Ingo Ott, Stefan Knapp, Laurent Meijer, Conrad Kunick

Supporting information

S1 Table: Results from a screening campaign[a] towards new CLK inhibitors. Residual enzyme activity [% ± SEM] in the presence of 6,7-dihydropyrrolo[3,4-*g*]indol-8(1*H*)-ones at 10 µM concentration[b]

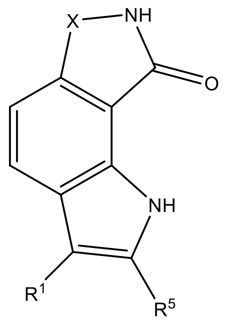

8a, S18a-e X: CH_2
S19 X: C=O

	R^1	R^5	CDK1/ cyclin B	CDK2/ cyclin A	CDK5/ p25	CDK9/ cyclin T	CK1	CLK 1	CLK 2	CLK 3	CLK 4	DYRK 1A	DYRK 1B	DYRK 2	DYRK 3	GSK3
**8a**	Phe	H	71±21	101±6	107±20	85±5	91±4	**8±2**	**14±3**	78±9	**2±0**	61±12	51±9	69±15	102±13	111±19
**S18a**	CH_3	C_2H_5	94±13	99±8	100±9	93±5	50±6	**20±2**	31±4	92±12	**7±2**	54±1	46±8	42±12	65±9	97±3
**S18b**	CH_3	CH_3	93±26	103±2	111±18	104±7	75±3	92±8	63±17	104±14	48±6	82±5	77±11	61±4	89±8	135±18
**S18c**	CH_3	Ph	77±26	109±8	105±11	89±6	57±5	**29±4**	**30±1**	93±9	**10±0**	58±7	50±7	57±25	91±17	112±2
**S18d**	C_2H_5	Ph	109±30	104±2	112±11	100±10	86±3	66±3	66±5	110±16	38±3	74±14	65±8	50±6	87±5	111±7
**S18e**	CH_3	4-CH_3-Ph	100±20	102±9	114±11	101±3	84±7	105±12	77±6	105±10	64±9	99±16	85±7	75±11	92±9	120±31
**S19**	CH_3	CH_3	81±27	108±8	94±4	90±5	65±5	52±10	33±7	91±11	**21±1**	41±8	39±5	**20±5**	57±3	51±5

[a] Compounds **8a**, **S18a-e** and **S19** were part of an in-house inventory.

[b] SEM = standard error of the mean
