# Supplementary material for: Molecular structures of cdc2-like kinases in complex with a new inhibitor chemotype
Source: PLoS One. 2018 May 3;13(5):e0196761. doi: 10.1371/journal.pone.0196761 (PMC5933782; doi:10.1371/journal.pone.0196761)
Supplement: S2 Table — (PDF) [file pone.0196761.s003.pdf]

Molecular structures of cdc2-like kinases in complex with a new inhibitor chemotype

Anne Walter, Apirat Chaikuad, Renate Helmer, Nadège Loaëc, Lutz Preu, Ingo Ott, Stefan Knapp, Laurent Meijer, Conrad Kunick

Supporting information

S2 Table: Results with compound KuWal151 (8c) in a crystal violet proliferation assay [a]

Cell line	GI_{50} [µM] ± SD
HT-29	0.12±0.01
MCF-7 [b]	0.35±0.16
MDA-MB-231	0.28±0.05
RC-124	0.44±0.10

[a] experiments carried out in triplicate, SD = sample standard deviation

[b] experiment carried out in duplicate
